# Amyloid β—Cholesterol Interplay: Removal of Cholesterol From the Membranes to Catalyze Aggregation and Amyloid Pathology

**DOI:** 10.1111/jnc.70380

**Published:** 2026-02-10

**Authors:** Rishiram Baral, Ruan van Deventer, Yuri L. Lyubchenko

**Affiliations:** ^1^ Department of Pharmaceutical Sciences University of Nebraska Medical Center Omaha Nebraska USA

**Keywords:** AFM, Alzheimer's disease, amyloid beta, Aβ42, cholesterol, membranes, protein aggregation

## Abstract

The interplay between the cholesterol metabolism and assembly of Aβ42 (the 42‐residue form of the amyloid‐β peptide) peptides in pathological aggregates is considered one of the major molecular mechanisms in the development of Alzheimer's disease (AD). Numerous in vitro studies led to the finding that high cholesterol levels in membranes accelerate the production of Aβ aggregates. The molecular mechanisms explaining how cholesterol localized inside the membrane bilayer catalyzes the assembly of Aβ aggregates above the membrane remain unknown. We addressed this problem by combining different AFM modalities, including imaging and force spectroscopy, with fluorescence spectroscopy. Our combined studies revealed that Aβ42 was capable of removing cholesterol from the membrane. Importantly, physiologically low concentrations of Aβ42 demonstrate such ability. Extracted cholesterol interacts with Aβ42 and accelerates its on‐membrane aggregation, which is a molecular mechanism explaining how cholesterol embedded in the membrane accelerates Aβ42 aggregation. The discovered ability of Aβ42 to remove cholesterol from membranes resulted in three major AD‐related events. First, free cholesterol catalyzes the assembly of Aβ42 in aggregates, which is the mechanism by which physiologically important Aβ42 monomers are converted into their pathological form. Second, the release of cholesterol from membranes leads to its accumulation in the brain, which is one of the risk factors associated with disease development and progression. Third, cholesterol depletion decreases membrane stiffness, which can result in deterioration of the function of membrane‐bound proteins, such as dendritic spine degeneration and, ultimately, synapse loss, a common pathological feature of AD.

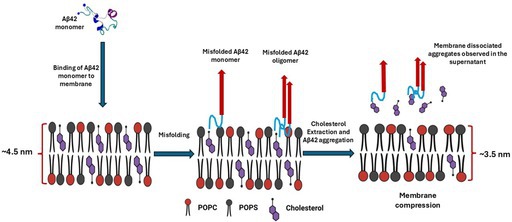

## Graphical abstract

The interplay between the cholesterol metabolism and assembly of Aβ42 (Amyloid beta‐42) peptide in pathological aggregates is considered one of the major molecular mechanisms in the development of Alzheimer's disease (AD). Our combined studies revealed that Aβ42 was capable of removing cholesterol from the membrane at physiologically relevant low concentrations. Extracted cholesterol interacts with Aβ42 and accelerates its on‐membrane aggregation. The discovered ability of Aβ42 to remove cholesterol from membranes, in addition to the assembly of Aβ42 aggregates, results in the accumulation of cholesterol in the brain and a decrease in membrane stiffness, which are the major risk factors associated with AD development and progression.

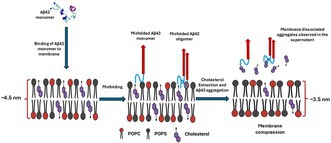

**Abbreviations**
25‐NBD: 25‐[N‐[(7‐nitro‐2‐1 3‐benzoxadiazol‐4‐yl)methyl]amino]‐27‐norcholesterol
AD: Alzheimer's disease
AFM: atomic force microscopy
Aβ42: amyloid beta‐protein 42
DMSO: dimethyl sulfoxide
HFIP: hexafluoroisopropanol
MD: molecular dynamics
MβCD: methyl‐beta cyclodextrin
POPC: 1‐palmitoyl‐2‐oleoyl‐glycero‐3‐phosphocholine
POPS: 1‐palmitoyl‐2‐oleoyl‐sn‐glycero‐3‐phospho‐L‐serine
RMS: root‐mean‐square
SLB: supported lipid bilayer

## 1 Introduction

The age‐related formation and accumulation of Amyloid beta (Aβ) aggregates in the extracellular space of the brain, forming plaques is a characteristic hallmark of Alzheimer's disease (AD) (Bate and Williams 2018; Friedrich et al. 2010). Elucidating the molecular mechanisms by which intrinsic and extrinsic factors influence the aggregation of Aβ42 is critical for devising effective therapeutic interventions that target and inhibit its self‐assembly (Lansbury and Lashuel 2006; Necula et al. 2007). Physiological concentration of Aβ42 in the low nanomolar level is the factor preventing the assembly of Aβ aggregates in test tubes, yet lipid membranes can affect the aggregation of Aβ (Henry et al. 2015; Rangachari et al. 2018; Williams and Serpell 2011). We have shown that membranes are capable of catalyzing Aβ aggregation at physiologically relevant low nanomolar concentrations of the peptide, further supporting a critical role of cellular membranes in the formation of pathological amyloid aggregates (Banerjee et al. 2017; Pan et al. 2019). This is the *on‐surface aggregation* pathway allowing for a spontaneous aggregation of Aβ peptides of different sizes and α‐synuclein protein at the nanomolar concentration range (Banerjee et al. 2017). The process takes place at ambient conditions, physiological pH values, and without additional mechanical stimulation of aggregation. We developed a theoretical model for surface‐mediated catalysis and tested the model in experiments (Pan et al. 2019). According to the model, aggregation starts with protein monomers transiently attaching to the surface due to molecular interactions. This process increases the local concentration of proteins, which in turn increases the probability of oligomerization reactions occuring on the surface. Of note, the catalytic effect of surfaces in amyloid aggregation explains the experiments on aggregation of Aβ40 in cell culture at low nanomolar concentrations (Podlisny et al. 1998). Our results are in line with (Lindberg et al. 2017), who reported catalytic properties of the zwitterionic lipid vesicles during the formation of Aβ42 fibrils (reviewed in (Cebecauer et al. 2017; Gorbenko and Kinnunen 2006; Khondker et al. 2017; Korshavn et al. 2017)).

The membrane composition is an important factor contributing to the on‐membrane aggregation of Aβ42, which was documented by various methods. Note a study (Habchi et al. 2018) in which the role of cholesterol in accelerating Aβ42 aggregation in the presence of vesicles composed of cholesterol and other phospholipids was demonstrated. The lipid metabolism and alteration of lipid composition in the brain are strongly associated with the pathogenesis of AD (He et al. 2025).

Cholesterol is a fundamental constituent of cellular membranes and plays a pivotal role in the nervous system (Feringa and Van der Kant 2021). The brain, as one of the organs with the richest cholesterol content in the human body, accounts for only 2% of total body weight but contains 25% of total body cholesterol (Dietschy and Turley 2001; Martín et al. 2014; Zhang and Liu 2015). Alterations in brain cholesterol metabolism are related to AD and other neurodegenerative diseases (Grao‐Cruces et al. 2023; Yang et al. 2022). The reduction in the efficiency of lipid transport mechanisms with age can lead to lipid imbalances and contribute to neurodegeneration (Di Paolo and Kim 2011). Suppression of cholesterol synthesis in astrocytes substantially reduces Aβ42 aggregation. Initial investigations into AD's lipid‐related mechanisms have revealed disrupted cholesterol movement from astrocytes to neurons, clearly showing the interrelationship between cholesterol homeostasis and AD (Di Paolo and Kim 2011). Evidence from the literature links AD pathogenesis with an increased concentration of cholesterol (Friedrich et al. 2010; Habchi et al. 2018; Wingo et al. 2019). It was demonstrated that membrane cholesterol content plays a key role in the neurotoxicity of Aβ‐amyloid (Abramov et al. 2011).

The membrane composition is the factor defining the catalytic property of the membrane, and the membrane‐bound cholesterol significantly accelerates the Aβ42 aggregation at physiologically relevant low nanomolar protein concentration (Banerjee et al. 2021; Hashemi et al. 2022). The Aβ42 aggregation on vesicles containing cholesterol revealed that the kinetics of aggregation depend on the cholesterol concentrations (Banerjee et al. 2021; Hashemi et al. 2022), but the molecular mechanism explaining how cholesterol in membranes facilitates aggregation of amyloids above the membrane remains unknown.

Here, we aim to unravel this mechanism by thoroughly characterizing the interaction between Aβ42 and cholesterol‐containing membranes. The studies with the use of various AFM modalities and fluorescence experiments revealed that Aβ42 is capable of extracting cholesterol from membranes, which leads to accelerated aggregation. The implication of this phenomenon to understand the interplay between the cholesterol homeostasis, membrane stiffness, and Aβ42 pathology is discussed.

## 2 Materials and Methods

### 2.1 Materials

1‐palmitoyl‐2‐oleoyl‐sn‐glycero‐s3‐phosphocholine (POPC) (cat. no. 850457C), 1‐palmitoyl‐2‐oleoyl‐sn‐glycero‐3‐phospho‐L‐serine, (POPS) (cat. no. 840034C) and 25‐[N‐[(7‐nitro‐2‐1,3‐benzoxadiazol‐4‐yl)methyl]amino‐27‐norcholesterol, (25‐NBD Cholesterol) (cat. no. 810250C) were purchased from Avanti Polar Lipids (Corda International Plc. Alabama, USA). Lyophilized cholesterol (cat. no. 700000P) and Methyl‐beta cyclodextrine (MβCD) (cat. no. M7439) were obtained from Sigma‐Aldrich (Sigma‐Aldrich Inc., St. Louis, USA). Lyophilized Aβ42 (cat. no. RP10017‐1) was procured from Genscript (Genscript, New Jersey, USA). Chloroform (cat. no. C2432) was procured from Sigma‐Aldrich Inc. The buffer solution used in this study is 10 mM HEPES, pH 7.4, with 150 mM NaCl and 10 mM CaCl_2_. In liquid AFM probes, MSNL‐10 were purchased from Bruker (Bruker, California, USA).

### 2.2 Preparation of Aβ42 Protein Solution

Stock solution of Aβ42 was prepared as described in our previous publications (Banerjee et al. 2017, 2021). Briefly, lyophilized Aβ42 powder (Genscript) was dissolved and sonicated for 5 min in 100 μL 1,1,1,3,3,3 Hexafluoroisopropanol (HFIP), maintaining the final concentration of 50 μM to break down pre‐formed oligomers. The formed thin film after evaporation of HFIP overnight, was reconstituted in DMSO, preserving the original concentration. This stock solution was stored at −20°C until use. An aliquot of the stock solution was dialyzed against 10 mM HEPES buffer (pH 7.4) and the final concentration was measured using NanoDrop at 280 nm. The dialyzed solution was then diluted to 50 nM using 10 mM HEPES buffer (pH 7.4) containing 150 mM NaCl and 10 mM CaCl_2_.

### 2.3 Assembly of 0.25 mg/mL POPC:POPS Supported Lipid Bilayers With Different Concentrations of Cholesterol

Stock solution of 10 mg/mL POPC, POPS, and cholesterol were stored at −20°C. The experimental working solution was prepared as described in the references (Cheng and London 2009; Li and London 2020) with slight modifications. Stock solutions of POPC, POPS, and cholesterol were mixed, and chloroform was evaporated under nitrogen flow to form a thin film. The film was resuspended in 10 mM HEPES (pH 7.4) containing 150 mM NaCl and 10 mM CaCl_2_, with a final lipid concentration of 0.25 mg/mL. Cholesterol concentrations were adjusted to 10%, 20%, and 30% (mol %) in separate solutions. These working solutions were stored at 4°C and used at room temperature unless specified.

Supported lipid bilayers (SLB) were prepared following the methodology outlined in our previous publications (Banerjee et al. 2021; Banerjee and Lyubchenko 2022). Briefly, a piece of mica, used as a solid support, was glued to hydrophobic‐coated glass slides, and its surface was freshly cleaved before phospholipid incubation. The phospholipid solution with cholesterol was sonicated for 45 min and incubated on the mica surface in a saturation chamber at 60°C for 50 min. Solvent evaporation during heating was compensated by adding deionized water as needed. After incubation, the solution was replaced with 10 mM HEPES buffer (pH 7.4) containing 10 mM CaCl_2_ and 150 mM NaCl to remove unruptured vesicles. The sample was cooled to room temperature for 10 min before imaging.

### 2.4 Treatment of Assembled SLB With Methyl‐β‐Cyclodextrin (MβCD) to Deplete the Cholesterol Content

The depletion of cholesterol in the assembled SLB was performed as per the method described in the reference^34^ with slight modification. Briefly, MβCD was dissolved in 10 mM HEPES buffer (pH 7.4) to prepare a 100 mM stock solution, which was further diluted to 3 mM using buffer with 10 mM CaCl_2_ and 150 mM NaCl for bilayer stability. A POPC:POPS:20% cholesterol bilayer (0.25 mg/mL) was incubated with 150 μL of 3 mM MβCD for 30 min at room temperature. After incubation, MβCD was rinsed off, and the bilayer was treated with 50 nM Aβ42 in buffer with salt. Time‐lapse AFM imaging was performed to observe Aβ42 aggregation. Force spectroscopy was used to measure the bilayer stiffness and thickness. A control experiment was conducted without Aβ42.

### 2.5 Time‐Lapse AFM Imaging

Liquid imaging in AFM using buffer solution was carried out in tapping mode using the MFP‐3D instrument (Asylum Research, Santa Barbara, CA, USA). The cantilever “E” of MSNL probes (Bruker, Santa Barbara, CA, USA), having a nominal spring constant of 0.1 N/m and a typical resonance frequency of 7–9 kHz, was used for scanning the bilayer surface. Prior to use, the MSNL probes were treated with methanol for 30 min to minimize protein adsorption on the tip. This procedure reduces the risk of tip contamination, which could otherwise compromise force measurements and obscure genuine biomolecular interactions. The homogeneity and smoothness of the lipid bilayer surface, devoid of any unruptured vesicles was ensured at the beginning of each time lapse‐experiment by scanning the larger area of 10 × 10 μm. Then, a smooth bilayer surface of 1 × 1 μm area was selected, followed by the incubation of Aβ42 solution. AFM imaging on the MFP‐3D until 6 h was automated using the integrated MacroBuilder software. The software automatically allowed the parking of the cantilever in between each scan. With this, the number of scans was restricted to prevent damage to the bilayer, as repeated probing by the AFM tip is known to cause degradation.

### 2.6 Measurement of Young's Modulus Using AFM Force Spectroscopy

The Young's modulus of the bilayers was calculated following the method described previously (Gabbutt et al. 2019; van Deventer and Lyubchenko 2024). The quantitative determination of the stiffness of the bilayer was performed in contact mode using MFP‐3D instrument (Asylum Research, Santa Barbara, CA, USA). An MSNL‐E cantilever with a spherical silicon nitride tip was employed to gently probe the bilayer surface under a constant applied force of 200 pN, using an approach and retraction velocity of 400 nm/s. The cantilever employed had a nominal spring constant of 0.1 N/m. For the individual force‐measurements, the deflection sensitivity was calibrated and the true spring constant was determined through thermal tuning. For the initial data fitting, an apparent tip radius of 10 nm was assumed with a spherical indentor, with Poisson's ratios of 0.25 for the tip and 0.50 for the bilayer. The Young's modulus was fitted from force distance curve using DMT model (Derjaguin et al. 1975). Young's modulus value of each point of probing was derived from individual pixels and plotted as histogram. All measurements were conducted in three independent experimental replicates, and the resulting data are presented as mean ± mean absolute difference (MAD).

### 2.7 Measurement of the Thickness of the Bilayer Using Edge Height

0.25 mg/mL POPC:POPS:20% Chol SLB was assembled on freshly cleaved mica as described in method above. The formation of the bilayer was confirmed by measuring the height of the edge of the bilayer with horizontal cross‐section accompanied with the measurement of Young's modulus, using the constant force of 200 pN. Multiple horizontal cross‐sections were taken near the bilayer edge, and the height difference between the mica substrate and the bilayer surface was used to determine the bilayer thickness. The thickness data obtained were plotted as histogram and fitted with single peak Gaussian fit using Magicplot Pro 2.3.92. All measurements were conducted in two independent experimental replicates (*n* = 2).

### 2.8 Measurement of Fluorescence Intensity of 25‐NBD Cholesterol Released From the Assembled Bilayer

The release of the fluorescently labeled cholesterol from the 0.25 mg/mL bilayer was measured as described previously (Beseničar et al. 2008; Ishii et al. 2010; Ostašov et al. 2013) with few modifications. 0.25 mg/mL POPC:POPS bilayer incorporating 20 mol% of 25‐NBD‐cholesterol was prepared following the protocol as mentioned previously. After assembly, the bilayer was thoroughly rinsed with 10 mM HEPES buffer (pH 7.4) to eliminate unincorporated fluorescently labeled cholesterol. Subsequently, the bilayers were incubated under three experimental conditions: (1) 50 nM Aβ42, (2) 3 mM MβCD, or (3) control buffer containing 10 mM HEPES, 150 mM NaCl, and 10 mM CaCl_2_. Aliquots were collected hourly from the bulk solution above the bilayer, and fluorescence intensity was recorded using a Varian Cary Eclipse Fluorescence Spectrophotometer. Fluorescence measurements were conducted with an excitation wavelength of 450 nm and an emission scan range of 470–700 nm, with a peak emission observed at 544 nm. All measurements were conducted in three independent experimental replicates, and the resulting data are presented as mean ± MAD.

### 2.9 AFM Data Analysis

The AFM images of the lipid bilayer acquired using the MFP‐3D were flattened, visualized and analyzed using Gwyddion v2.66 (Gwyddion, Czech Republic). The formation of the phospholipid bilayer was confirmed by measuring the height of the bilayer from the mica surface up to the edge of the bilayer. The number of Aβ42 aggregates and their volumes were determined using the Enum Feature tool in FemtoScan software (Advanced Technologies Center, Moscow, Russia). The data generated from the AFM topographic and force spectroscopy experiments were plotted as histograms using Magic Plot software. When necessary, the data were fitted with a single‐peak Gaussian distribution. The error bars in histograms represent the standard deviation. To compare the results obtained in three independent experimental replicates, an approach was used in which averaged mean values were obtained from the mean values derived from individual experiments. The deviations of the mean values were calculated from the averaged value, which is termed mean absolute deviation (MAD).

### 2.10 Statistical Analysis

Statistical significance between different time points and experimental groups was assessed through student's *t*‐test using data analysis toolpak of Microsoft Excel 365. The sample size of *n* = 100 independent measurements per area was chosen to ensure statistical robustness for both AFM topographical analysis and force‐distance‐derived nanomechanical properties. In scanning probe microscopy, a sampling density on the order of ≥ 100 measurements is widely used as an empirical benchmark for achieving statistical convergence of ensemble‐averaged properties and for reducing relative sampling error to approximately the 5%–10% range for comparable distributions. This heuristic is well established in the nanoparticle and AFM metrology literature (Grobelny et al. 2009; Masuda and Gotoh 1999). Measurements were acquired across multiple spatially distinct areas and independent experimental replicates, ensuring that the reported size distributions and nanomechanical parameters reflect the global surface population rather than localized heterogeneities or measurement artifacts. Prior to any statistical comparisons, normality and equal variance (F‐test) were confirmed. None of the data points were excluded during the analysis. Statistical analysis was performed using unpaired two‐sample Student's *t*‐test under the assumption of unequal variances. The means of independent datasets measured at 0 h (*n* = 3) and 6 h (*n* = 3) were compared to determine whether these mean values differed significantly between groups. A two‐tailed test was used to detect differences in either direction, and results were considered statistically significant at *p* < 0.05. The complete statistical test report for all comparative analyses has been provided in the final section of the Supporting Information.

## 3 Results

### 3.1 Cholesterol in the Membrane Catalyzes Aβ42 Protein Aggregation

We assembled supported‐lipid bilayers composed of 0.25 mg/mL 1‐palmitoyl‐2‐oleoyl‐sn‐glycero‐3‐phospho‐L‐serine (16:0–18:1 POPS) and 1‐palmitoyl‐2‐oleoyl‐glycero‐3‐phosphocholine (16:0–18:1 POPC) in a 1:1 M ratio with different cholesterol concentrations. Solution of Aβ42 was placed above the bilayer, and Atomic Force Microscopy (AFM) imaging and force spectroscopy were applied to characterize aggregation of Aβ42 monomers and their effect on the bilayer. The experiments were conducted using 50 nM Aβ42 peptides, which are in the range of physiologically relevant concentrations.

Three different concentrations of cholesterol (10%, 20%, and 30%) were used to generate stable supported‐lipid bilayers with a topographically smooth morphology as described in the methods section. The formation of the bilayer was verified by measuring the thickness of the bilayer at the edge of a smooth area (white dashed line in Figure S1A) and the results from multiple measurements (*n* = 100) (Figure S1B) produce the mean value 4.23 ± 0.92 nm, which corresponds to the typical thickness of similar bilayers (Banerjee and Lyubchenko 2022; van Deventer and Lyubchenko 2024). The smoothness of the bilayers was monitored with AFM, by measuring the root‐mean‐square (RMS) roughness of the smooth area. The smoothness of the entire area produced mean values of 0.059 ± 0.002 nm, which is suitable for detecting the formation of amyloid aggregates (Banerjee et al. 2021; Banerjee and Lyubchenko 2022; van Deventer and Lyubchenko 2024). This parameter remains constant during the 6‐h observation time (Figure S1C, Table S1).

**FIGURE 1:**
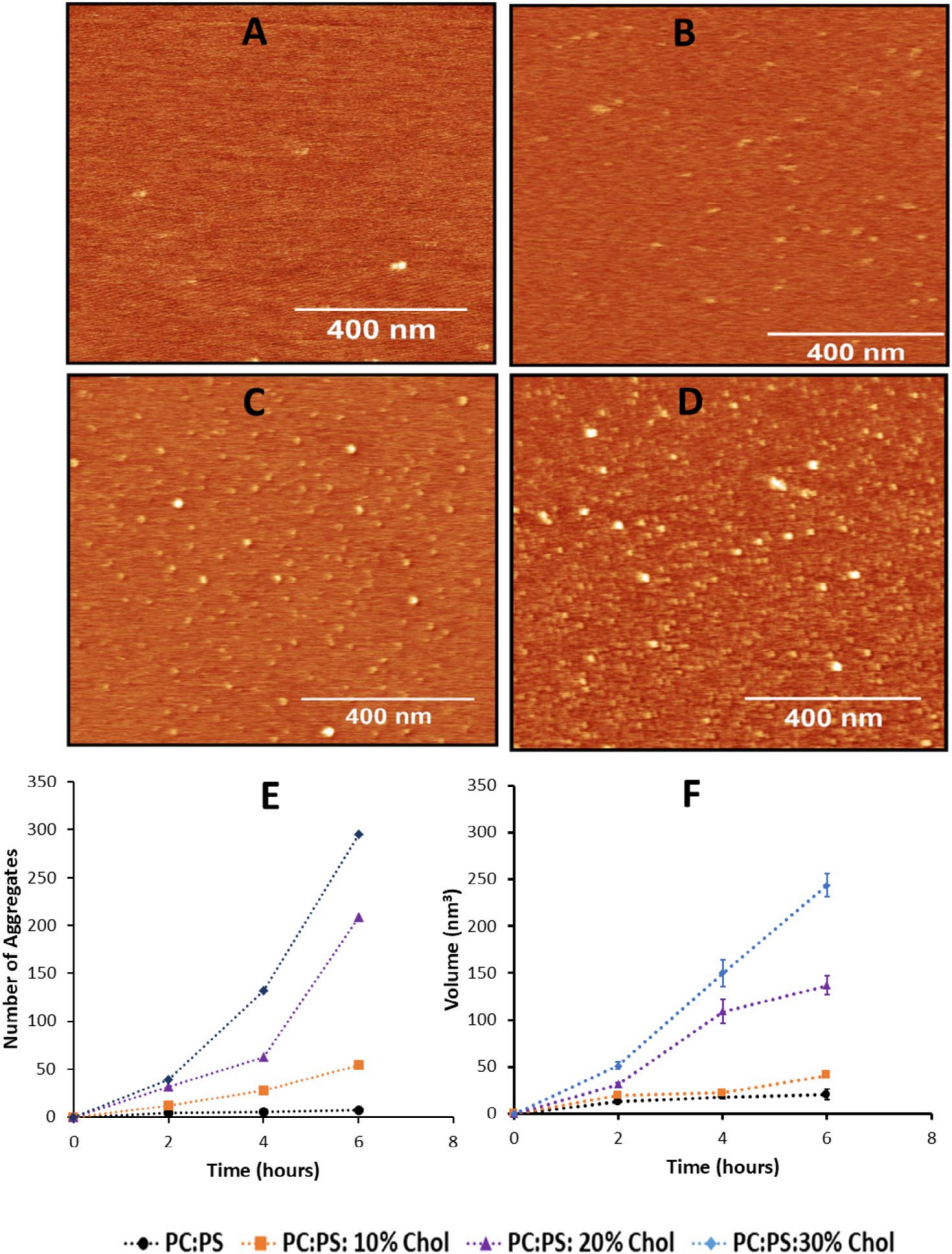
AFM data for aggregation of 50 nM Aβ42 on POPC: POPS bilayer depending on cholesterol in the bilayer. (A–D) AFM images of the aggregation of 50 nM Aβ42 on 0.25 mg/mL POPC:POPS lipid bilayer at 6 h (A) without cholesterol, (B) with 10% cholesterol, (C) with 20% cholesterol, (D) with 30% cholesterol. The scan size is 1 × 1 μm. (E) The average number of aggregates, (F) the average volume of these aggregates over time. The number of aggregates values represent numbers of aggregates in 1 × 1 μm scan of the same size; the volumes are the mean volume of the aggregates with error bars as MAD. AFM, Atomic Force Microscopy; Aβ42, Amyloid beta‐42; POPC, 1‐palmitoyl‐2‐oleoyl‐sn‐glycero‐3‐phospho‐L‐serine; POPS, 1‐palmitoyl‐2‐oleoyl‐sn‐glycero‐3‐phospho‐L‐serine; MAD, Mean Absolute Difference.

Once the bilayer was assembled, 50 nM Aβ42 solution in 10 mM HEPES buffer with 150 mM NaCl and 10 mM CaCl_2_ was added on top of the bilayer, and the assembly of aggregates was monitored with time‐lapse AFM imaging. We used 10 mM CaCl_2_, which is higher than physiological concentrations in the brain (Forsberg et al. 2019), this was motivated by its demonstrated ability to enhance phospholipid bilayer stability over extended timescales (Banerjee et al. 2021; Banerjee and Lyubchenko 2022), thereby enabling prolonged and reliable experimental measurements. The set of data characterizing the Aβ42 aggregation on bilayers without and with 10%, 20%, and 30% of cholesterol taken after 6 h of continuous observations is shown in Figure 1. Selected AFM images for 6 h of incubation with 50 nM Aβ42 solution on bilayers with 0%, 10%, 20%, and 30% cholesterol are shown in Figure 1A–D, respectively. These images demonstrate the increase in the number and sizes of aggregates as the cholesterol concentration increases. A set of additional AFM images corresponding to different times for all bilayers is displayed in the supplement (Figure S2). The aggregates appear as bright globular features in all bilayers, which is consistent with our previous paper (Banerjee et al. 2021; Hashemi et al. 2022). The number and sizes of aggregates were measured, the histograms for sizes (volumes) are shown in Figure S3 and the number of aggregates and their sizes as a function of time are plotted in Figure 1E,F, respectively. These graphs show that the number of aggregates and their sizes grow with the increasing cholesterol concentration as a function of time.

### 3.2 Aβ42 Reduces Membrane Stiffness

**FIGURE 2:**
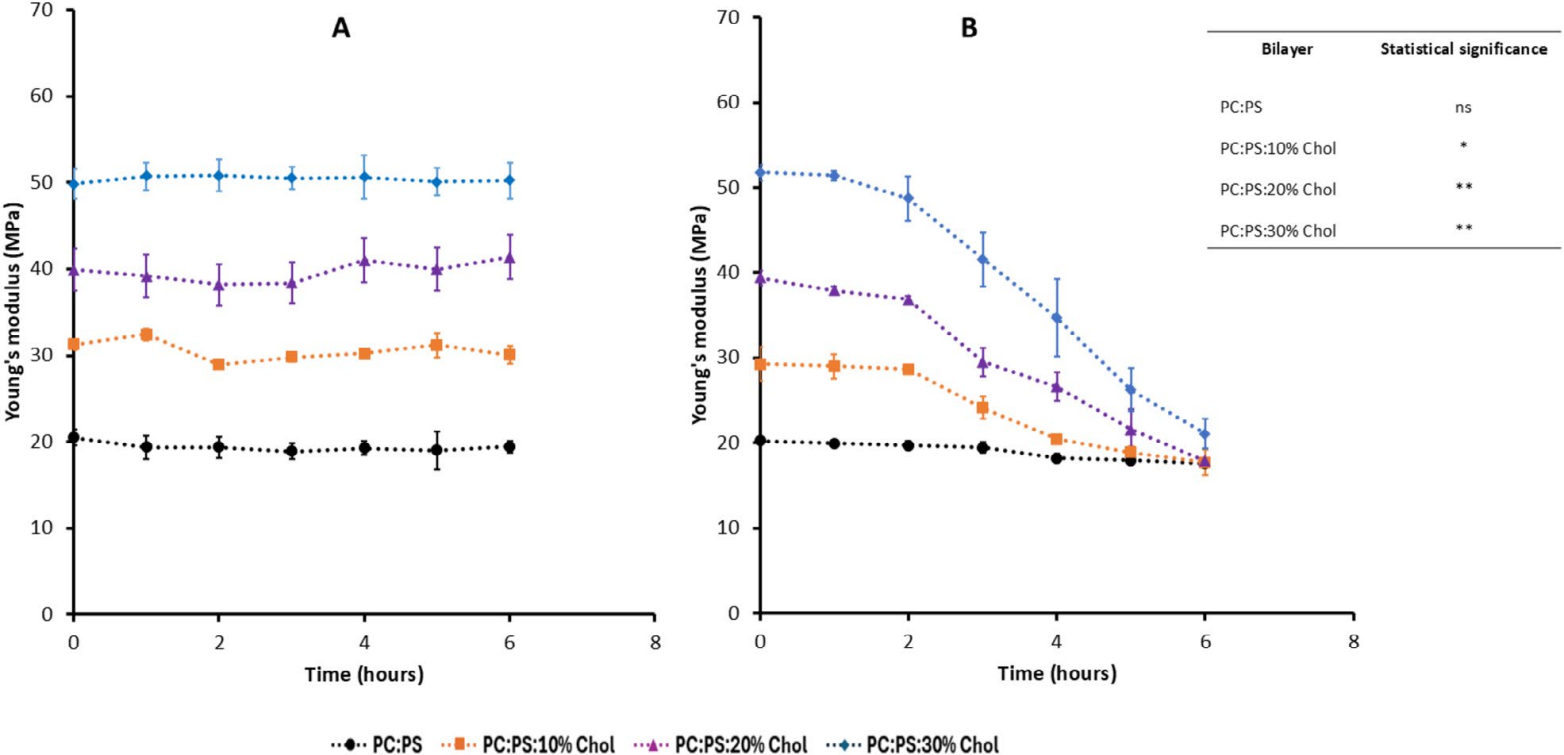
Young's modulus of POPC: POPS bilayer with different concentrations of cholesterol with and without 50 nM Aβ42 above the bilayer. All experiments were performed in triplicate (*n* = 3) with the data points taken from Tables S5 and S9. The graphs show the Young's modulus values for 0.25 mg/mL phospholipid bilayer with different mol% of cholesterol from 0 to 6 h as mean ± MAD. (A) Graphs for experiments in the absence of 50 nM Aβ42 (*n* = 100) made for data taken from Table S5. (B) The set of graphs for experiments in the presence of 50 nM Aβ42 from 0 to 6 h (*n* = 100). made for data taken from Table S9. The statistical significance between the initial time point (*t* = 0 h) and the final time point (*t* = 6 h) for each bilayer prepared with varying cholesterol concentrations in the presence of Aβ42 is presented in the inset table of Figure 2B. The difference in means between the different time points was tested using unpaired two‐tailed Student's T‐tests, with significance levels as *p < 0.05, **p < 0.01, ns = not significant. For PC:PS, PC:PS:10% Chol, PC:PS:20% Chol, PC:PS:30% Chol bilayer treated with 50 nM Aβ42, *p*‐value = 0.196, 0.027, 0.006 and 0.002, respectively.

We assessed how Aβ42 aggregation influences the mechanical properties of bilayers containing 10%, 20%, and 30% cholesterol. During this process, two parameters of the membrane were measured: stiffness and thickness. Stiffness was characterized by Young's modulus by performing multiple measurements every hour over the 6 h period at various locations on the surface, which was done in parallel with topographic imaging, ensuring that mechanical properties were recorded for the same set of AFM images. The respective histogram of Young's modulus values for different cholesterol concentrations in the absence of Aβ42 are shown in Figure S4. Similar histograms were obtained for other times, the histograms were approximated with Gaussians, the mean Young's modulus values for each hour for each type of bilayer were obtained and assembled in Table S2. The experiments were repeated, and the Young's modulus values for each experiment corresponding to cholesterol concentration 10%, 20% and 30% as a function of time are assembled in Tables S3 and S4. The values averaged over different experiments as a function of time for each cholesterol concentration are shown in Table S5. These averaged values are plotted in Figure 2A. Error bars on these graphs correspond to the variability of the mean values of each experiment (mean absolute deviation, MAD). The results demonstrate that cholesterol increases the initial stiffness of the bilayers, with the highest value observed at 30% cholesterol (Figure 2A, blue points). The stiffness of each type of bilayer remained stable within the entire observation period, 6 h.

Next, Aβ42 solution was placed on the bilayers with the same set of concentrations of cholesterol and Young's modulus were measured every hour over the 6 h period. Histograms for Young's modulus measurements corresponding to 0, 3, and 6 h are shown in Figure S5. Similar data were obtained for other time points; the histograms were approximated with Gaussians, mean values were obtained and assembled in Table S6. The histograms for Young's modulus measurements for bilayers with cholesterol were shifted towards lower values, which corresponds to the mean Young's modulus value without cholesterolwith the highest effect for the bilayer with 30% cholesterol. The experiments were repeated, the histograms for Young's modulus values measurements were obtained, the mean values were calculated and the data for additional two independent experiments are assembled in Tables S7 and S8. Mean values for each time point averaged over three experiments were calculated and assembled in Table S9. These values as a function of time for each cholesterol concentration were plotted in Figure 2B with error bars corresponding the variability of the Young's modulus mean values in experiments. These data demonstrate that upon incubation with Aβ42, Young's modulus values decrease over time for all bilayers, converging to similar values after 6 h. The strongest effect is for the bilayer with 30% cholesterol concentration (Figure 2B) in which the initial value 52 MPa drops after 6 h to ~20 MPa corresponding to the bilayer stiffness without cholesterol. Importantly, no change in Young's modulus were observed in control experiments with no added Aβ42 (Figure 2A). These findings suggest that in addition to forming aggregates on the bilayer surface, Aβ42 reduces bilayer stiffness.

**FIGURE 3:**
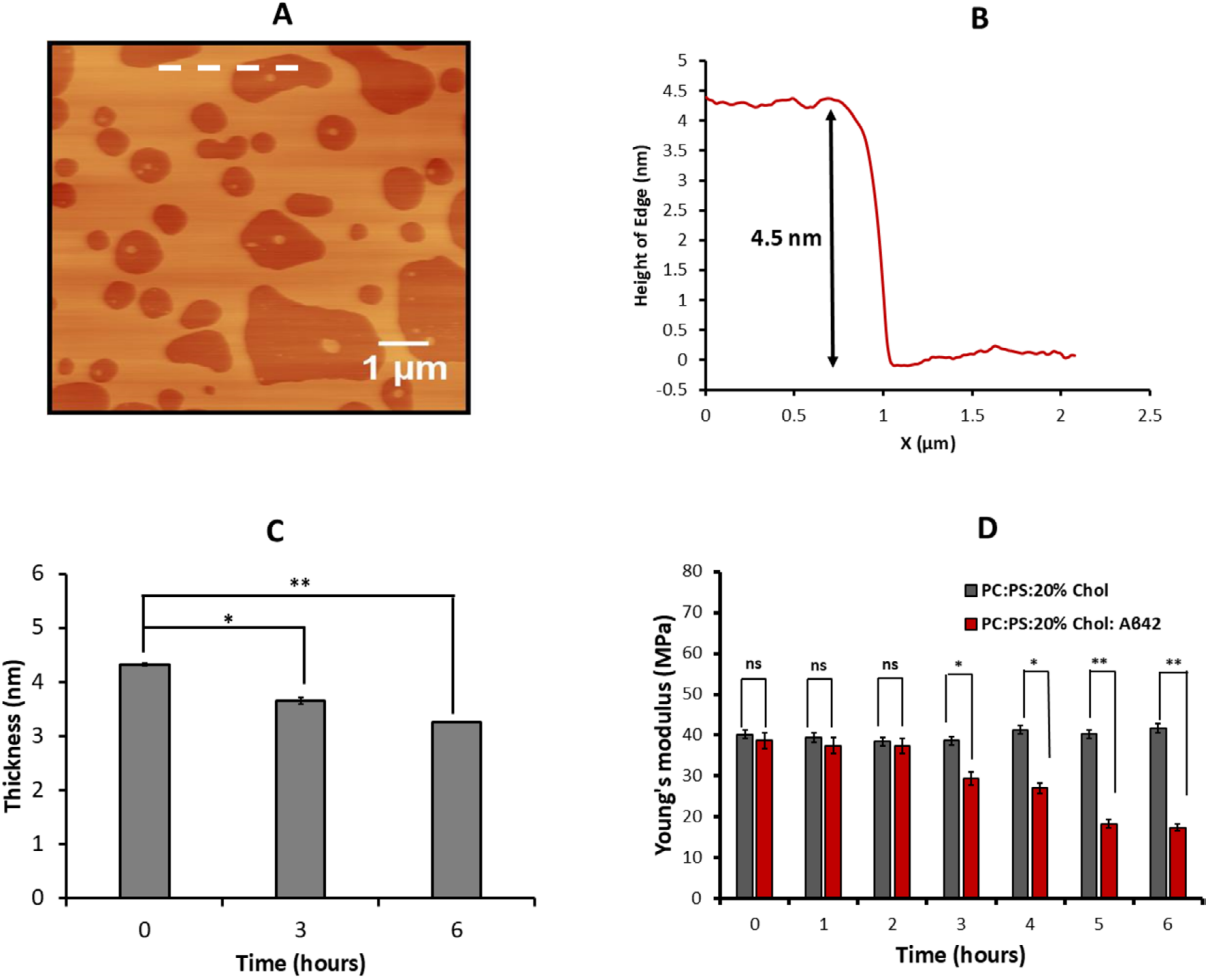
Thickness measurement for POPC:POPS bilayer with 20% Chol incubated with Aβ42 on top of the bilayer. (A) Representative AFM image of 0.25 mg/mL POPC:POPS:20% Chol phospholipid bilayer incubated with Aβ42 where the thickness was measured. The scan size is 7 × 7 μm. (B) The representative height traces correspond to the horizontal cross section shown by white line in A. (C) Average thickness of the membrane obtained from the several cross section from 0‐time point to 6 h. Multiple thickness values were measured at several areas within different region at specific time points, and 100 such points were approximated with Gaussians. The mean values obtained from the approximations, along with the mean absolute difference, are shown as bar graph. The difference between means between initial time points and final time points were tested using unpaired two‐tailed Student's *t*‐test, with significance levels as *p < 0.05, **p < 0.01 and ns = not significant. The differences observed at 3 and 6 h relative to the initial time point with *p*‐values = 0.04 and 0.007, respectively. (D) Young's modulus of 0.25 mg/mL phospholipid bilayer with 20% of cholesterol measured in parallel with the thickness measurements on the smooth surface shown in A from 0 to 6 h. Multiple Young's modulus values taken at various locations within the 1 × 1 μm surface were obtained and 100 such points were approximated with Gaussians. The mean values obtained from the approximations, along with the mean absolute difference, are shown as a bar graph. The difference between means between control and Aβ42 treated groups were tested using unpaired two‐tailed Student's *t*‐test, with significance levels as *p < 0.05, **p < 0.01, and ns = not significant. The difference observed between control and Aβ42 treated group at 0, 1, 2, 3, 4, 5, and 6 h are given as *p*‐value = 0.6121, 0.9164, 0.3753, 0.0305, 0.0238, 0.0031, 0.0024 respectively.

The AFM experiments with three cholesterol concentrations (10%, 20%, and 30%) were performed, which resulted in the hypothesis of cholesterol depletion from the bilayer. Subsequent analyses focusing on membrane thickness measurements and fluorescence studies were done with 20% cholesterol, which is within physiological range in the brain (20%–25% Cholesterol) (Dietschy and Turley 2001; Zhang and Liu 2015). Following the stiffness measurements, the time‐dependent changes in membrane thickness were evaluated. Multiple horizontal cross‐sections were taken near the bilayer edges, and the height difference between the mica substrate and the bilayer surface was used to determine the bilayer thickness. These measurements were performed in parallel with stiffness measurements for the POPS: POPC bilayers containing 20% cholesterol incubated with 50 nM Aβ42, where the results are shown in Figure 3. A representative topographic AFM image of the 7 × 7 μm area used for thickness measurements at various time points is shown in Figure 3A, illustrating the bilayer surface at 0 h. A corresponding horizontal cross‐section profile (indicated by the white line in Figure 3A) is presented in Figure 3B, showing the bilayer edge height at 0 h. Approximately 100 such horizontal cross‐sections were collected at multiple locations across the surface in several frames acquired at each time point. Thickness measurements for the bilayer incubated with 50 nM Aβ42 at different time points are compiled as histograms in Figure S6, along with the corresponding AFM images. The histograms were fitted with Gaussian functions, and the extracted mean values are reported. The height difference between the mica substrate and the bilayer corresponds to an initial thickness of ~5 nm. Average thickness values obtained from two independent experiments are summarized in Table S10, together with the respective means and mean absolute differences. These values are presented as bar plots in Figure 3C, illustrating the measurable changes in bilayer thickness over the 6‐h observation period.

Figure S7 shows bilayer thickness measurements from the initial time point through 6 h for the 20% cholesterol bilayer in the absence of Aβ42 (control), along with the corresponding AFM images. No detectable change in bilayer thickness was observed under these conditions. Average thickness values from two independent experiments are summarized in Table S11, together with the corresponding means and mean absolute differences.

Parallel to these measurements, in the same set of bilayers, Young's modulus measurements were made using the methodology described above. The histograms for the measurements are approximated with Gaussians as shown in Figure S8. The data illustrate the decrease of the Young's modulus from 38.59 ± 1.84 MPa at 0 time to 17.70 ± 0.84 MPa at 6 h. The experiments were repeated, and averaged values for Young's modulus for three independent experiments are presented in Table S12 along with the mean values calculated by averaging over three experiments. Similar analysis was done for the control with no Aβ42 added and the results are shown in Table S13. These values as vertical bars are plotted in Figure 3D. Red bars correspond to the measurements for bilayers with Aβ42 and black bars show the results of the control experiment—no Aβ42. Similar to the data in Figure 2B, the red bars values decrease with the incubation time reaching after 6 h value close to the one for POPC/POPS bilayer without cholesterol. Thus, the presence of Aβ42 above the bilayers reduces their stiffness and causes a measurable decrease in bilayer thickness. As an additional control, thickness measurements were performed on POPC:POPS bilayers lacking cholesterol. These bilayers were relatively thinner (~3.5 nm), and their thickness remained largely unchanged in both the presence (Figure S9, Table S14) and absence of Aβ42 (Figure S10, Table S15). Note that our studies were performed at the nanomolar range of Aβ42. Experiments with higher concentrations of Aβ peptides can lead to changes in the bilayer thickness including the formation of pores (Bhatia et al. 2000; Kayed et al. 2004). According to (Bode et al. 2017) no damage to membranes occurs within nanomolar Aβ concentrations. We did observe local indentations of the bilayer but assembled with lower concentrations of phospholipids, although similar to the current studies no changes in the bilayer topography were observed with a traditional concentration of phospholipids (van Deventer and Lyubchenko 2024).

### 3.3 Depleting Cholesterol From Membranes

We hypothesize that the drop in bilayer stiffness and thickness to values similar to those without cholesterol is due to the removal of cholesterol. To test this hypothesis, we conducted force spectroscopy studies using methyl β‐cyclodextrin (M*β*CD), a known cholesterol‐extracting agent (Beseničar et al. 2008; Mahammad and Parmryd 2015).

**FIGURE 4:**
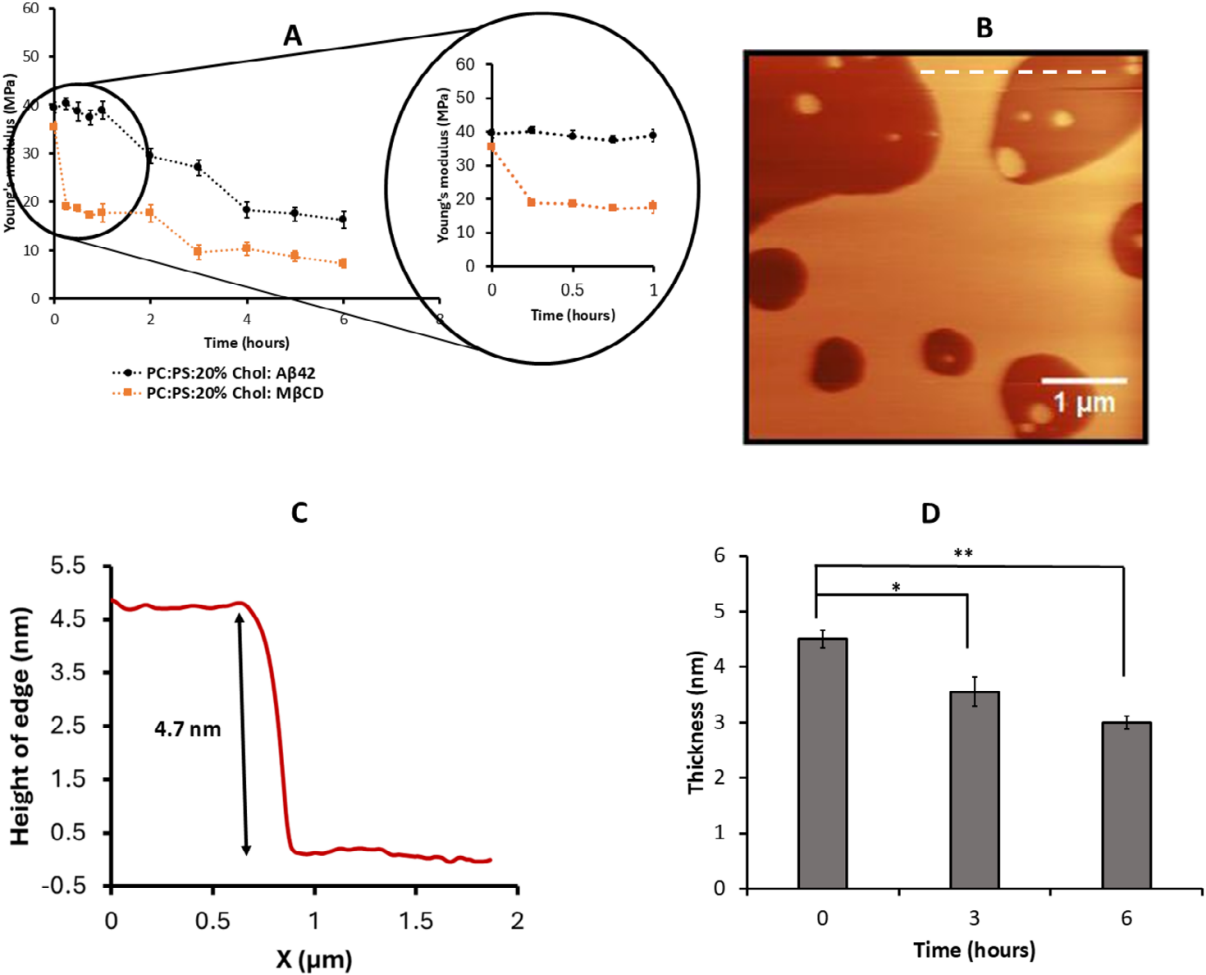
Young's modulus and thickness measurement for POPC:POPS with 20% Chol bilayer incubated with MβCD on top of the bilayer (A) Young's modulus of 0.25 mg/mL phospholipid bilayer with 50 nM Aβ42 (black dots) and 3 mM MβCD (orange dots) (*n* = 100). The zoomed circle on the right illustrates the kinetics for the 1 h of observation. (B) Representative AFM image of 0.25 mg/mL POPC:POPS:20% Chol phospholipid bilayer incubated with MβCD where the thickness was measured. The scan size is 5 × 5 μm. (C) The representative height traces correspond to the horizontal cross section shown by white line in B. (D) Average thickness of the membrane obtained from the several cross section from 0‐time point to 6 h. Multiple thickness values were measured at several areas within different region at specific time points, and 100 such points were approximated with Gaussians. The mean values obtained from the approximations, along with the mean absolute difference, are shown as bar graph. The difference in means between initial time point and final time points were tested using unpaired two‐tailed Student's *t*‐test, with significance levels as *p < 0.05, **p < 0.01 and ns = not significant. The differences observed at 3 h and 6 h relative to the initial time point with *p*‐values = 0.042 and 0.009, respectively. MβCD; Methyl‐β cyclo‐dextrine.

A solution of MβCD (3 mM in 10 mM HEPES buffer (pH 7.4) containing 150 mM NaCl and 50 mM CaCl_2_) was placed on the top of a POPC:POPS bilayer with 20% cholesterol and changes in Young's modulus were monitored for a time period over 6 h. The mean Young's modulus values are shown in (Figure 4A, orange data points), with the error bars indicating the MAD. The average Young's modulus values obtained from three independent experiments in the presence of MβCD are presented in Table S16, along with the corresponding mean and MAD values. Within the first 15 min of MβCD exposure, the bilayer stiffness decreased from 37.18 ± 1.19 MPa to 10.52 ± 0.84 MPa, (shown in zoomed circle of Figure 4A) with a gradual decrease over the entire observation period. The final Young's modulus value matches the stiffness of the POPC: POPS without cholesterol (Figure 2A, Table S2).

The bilayer thickness was measured in conjunction with Young's modulus. A representative topographic AFM image of the 5 × 5 μm region used for thickness measurements at various time points is shown in Figure 4B, illustrating the bilayer surface at 0 h. A corresponding horizontal cross‐section profile (indicated by the white line in Figure 4B) is presented in Figure 4C, showing the initial bilayer edge height at 0 h. Additional data are provided in Figure S11. Mean thickness values were calculated and are plotted in Figure 4D, demonstrating that changes in bilayer thickness occur concurrently with alterations in bilayer stiffness. The experiments in the presence of MβCD were repeated, and averaged thickness values obtained from two independent experiments are presented in Table S17, along with the corresponding mean values and the errors as MAD values. These are graphically shown as vertical bars in Figure 4D which shows measurable change in the bilayer thickness over 6 h observation.

For direct comparison of the data above, we repeated the experiment for a similar bilayer placing 50 nM Aβ42 above it. The data in (Figure 4A, in black symbols) show the gradual decrease in Young's modulus. Comparing two sets of data (Figure 4A) reveals that MβCD and Aβ42 decrease the stiffness of the bilayer. Importantly, MβCD and Aβ42 reduce the bilayer stiffness to levels comparable to cholesterol‐free membranes, suggesting that Aβ42 is analogous to that of MβCD deplete cholesterol from the bilayer.

### 3.4 Extraction of Cholesterol From the Membrane by Aβ42 and MβCD: Fluorescence Spectroscopy Studies

To directly test the hypothesis on the cholesterol extraction by Aβ42 and detect free cholesterol depleted from the membrane, we conducted experiments using fluorescently labeled cholesterol incorporated into a POPC:POPS bilayer. If cholesterol is released into the solution above the bilayer, it can be detected via fluorescence measurements of the solution above the membrane. We used 25‐[N‐[(7‐nitro‐2‐1,3‐benzoxadiazol‐4‐yl)methyl]amino‐27‐norcholesterol (25‐NBD cholesterol), a common cholesterol analog, in fluorescence studies on membranes (Robalo et al. 2013).

**FIGURE 5:**
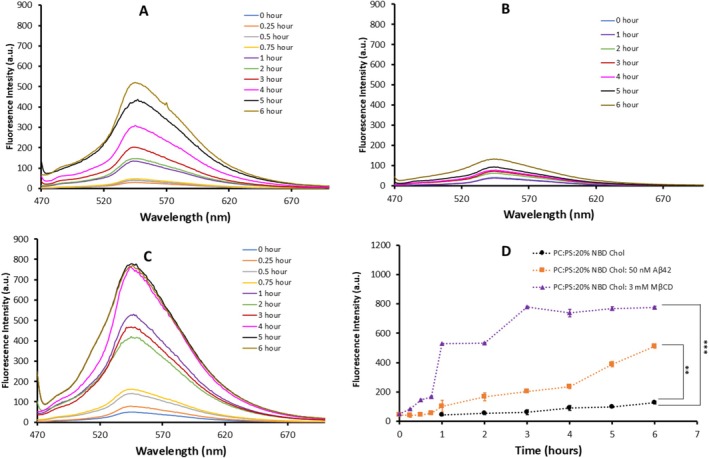
Fluorescence intensity measurements of solution above POPC: POPS bilayers containing 20 mol% fluorescently labeled cholesterol (25‐NBD cholesterol). The temporal changes in fluorescence intensity were monitored in solutions collected from POPC: POPS bilayers (0.25 mg/mL) incorporated with 20% 25‐NBD cholesterol under the following conditions: (A) incubation with 50 nM Aβ42, (B) exposure to 10 mM HEPES buffer with salt, (C) treatment with 3 mM MβCD and (D) the graphs of maxima in florescence emission spectra measure after averaging over three independent experiments (Tables S15–S17). Excitation of the fluorophore was done at 450 nm, and emission spectra were recorded from 470 nm to 700 nm, with a peak emission observed at 544 nm. The difference between means of the groups were tested using unpaired two‐tailed Student's T‐tests with significance levels as *p < 0.5, **p < 0.005, ***p < 0.001, ns = not significant. The differences observed at 6 h of Aβ42 and MβCD treated group with control is *p* = 0.00428 and 0.00094 respectively. 25‐NBD cholesterol; 25‐[N‐[(7‐nitro‐2‐1,3‐benzoxadiazol‐4‐yl)methyl]amino‐27‐norcholesterol.

A POPC: POPS bilayer containing 20% 25‐NBD cholesterol was assembled, and 50 nM Aβ42 was placed on the top of the membrane. Aliquots of the solution above the membrane were collected and fluorescence spectra were measured. The set of spectra measured over time shown in Figure 5A demonstrate the increase of fluorescence in the solution and the effect is higher for longer incubation time. Control experiments with the buffer above the bilayer without Aβ42 in Figure 5B shows some increase of fluorescence that can be attributed to the spontaneous release of weakly incorporated cholesterol from the bilayer. Thus, there is a dramatic difference in the fluorescence values for experiments with Aβ42 compared with the control. An apparent increase in intensity of fluorescence spectra over time suggests cholesterol accumulation above the membrane. Similar experiments were performed with MβCD and the results of the fluorescence measurements are shown in Figure 5C. There is a time‐dependent increase of fluorescence due to the accumulation of 25‐NBD cholesterol in the bulk solution above the membrane.

The experiments were repeated, the maxima values of fluorescence at each time point were measured and the data for experiments with Aβ42, MβCD and the control are shown in Tables S18–S20, respectively. Along with fluorescence values for each experiment, the mean values were calculated, and they are shown as last columns in each table. These mean values for Aβ42, MβCD and the control as a function of time are plotted in Figure 5D. Error bars correspond to the MAD of fluorescence values calculated from their variability in the three experiments. The graphs show that accumulation of cholesterol occurs in the presence of Aβ42 and MβCD although the rate is faster for MβCD. However, this difference should be considered in the context of the nearly five orders of magnitude lower Aβ42 concentration compared with that of MβCD used in these experiments. Importantly, the plateau level in the fluorescence values for Aβ42 was 70% of that for MβCD regardless of a considerably lower concentration of Aβ42.

### 3.5 AFM Topographic and Force Spectroscopy Studies of Aβ42 Interaction With POPC:POPS Bilayer Containing Fluorescently Labeled 25‐NBD Cholesterol

**FIGURE 6:**
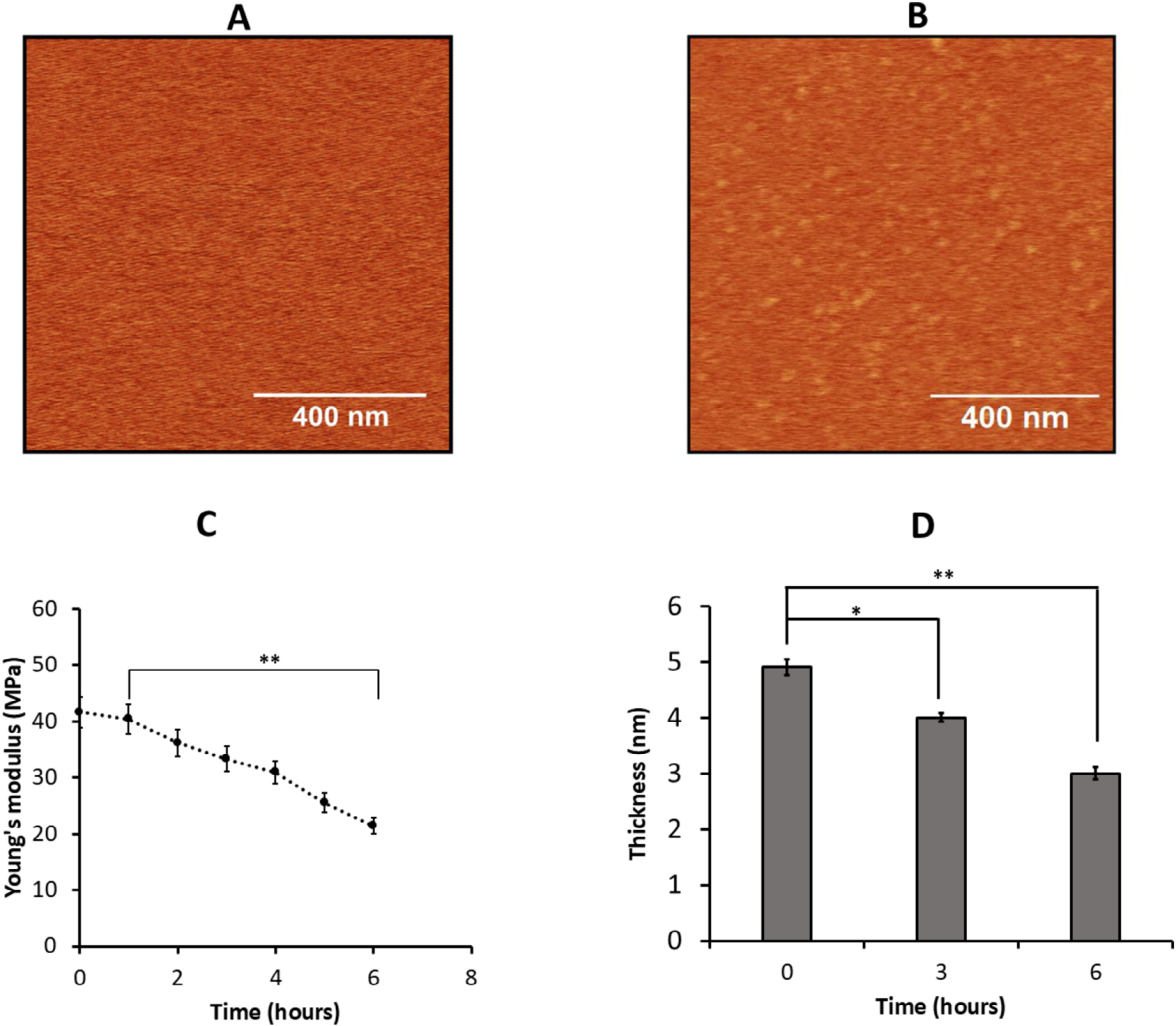
AFM‐time lapse imaging and mechanical characterization of bilayer with 25‐NBD cholesterol. AFM imaging was performed on a 0.25 mg/mL POPC:POPS phospholipid bilayer incorporating 20% 25‐NBD cholesterol in the presence of 50 nM Aβ42, with scans acquired at (A) 0 h and (B) 6 h. The scan area was set to 1 × 1 μm. (C) Young's modulus of the phospholipid bilayer under these conditions was determined (*n* = 100). The difference between means of the initial time point and final time point was tested using unpaired two‐tailed Student's T‐tests with significance levels as *p < 0.5, **p < 0.005, ***p < 0.001, ns = not significant. The differences in Young's modulus between 1 and 6 h observed was *p* = 0.00412. (D) Average thickness of the membrane obtained from the several cross sections from 0‐time point to 6 h. Multiple thickness values were measured at several areas within different regions at specific time points, and 100 such points were approximated with Gaussians. The mean values obtained from the approximations, along with the mean absolute difference, are shown as a bar graph. The difference between means of the initial time point and final time point was tested using unpaired two‐tailed Student's *t*‐tests with significance levels as *p < 0.5, **p < 0.005, ***p < 0.001, ns = not significant. The differences observed at 3 h and 6 h relative to the initial time point with *p*‐values = 0.0485 and 0.0092, respectively.

Although 25‐NBD cholesterol is commonly used as a cholesterol analog in fluorescence measurements, potential differences between fluorophore‐labeled and native cholesterol cannot be excluded. To assess whether the fluorophore moiety affects Aβ42 aggregation on the membrane, we assembled a 0.25 mg/mL POPC:POPS bilayer containing 20% 25‐NBD cholesterol and monitored the aggregation of 50 nM Aβ42 on its surface using AFM for up to 6 h. Images taken at 6 h (Figure 6B) confirmed aggregate formation on the bilayer compared to the initial time point (Figure 6A).

Young's modulus measurements (Figure 6C) illustrate the decrease of stiffness from 41.63 ± 2.73 MPa to 21.43 ± 1.40 MPa, closely aligning with values obtained for the POPC: POPS bilayer containing cholesterol (Figure 2B). Table S21 presents the average Young's modulus values obtained from three independent experiments in bilayers containing 20% 25‐NBD cholesterol incubated with 50 nM Aβ42, together with the corresponding means and MAD values as errors. Bilayer thickness (Figure 6D) decreased substantially following Aβ42 incubation, reaching values similar to those observed in cholesterol‐free bilayers. Detailed thickness measurements for bilayers containing 20% 25‐NBD cholesterol exposed to 50 nM Aβ42 are presented in Figure S12. The average thickness values obtained from two independent experiments in bilayer with 20% 25‐NBD cholesterol incubated with 50 nM Aβ42 are presented in Table S22, along with the corresponding mean values.

## 4 Discussion

The results described above revealed novel features of the interaction of Aβ42 with the cholesterol containing phospholipid bilayers. The topographic AFM studies directly visualized aggregation of Aβ42 on the surface and the process is accelerated by cholesterol (Figure 1), which is in line with our previous finding (Banerjee et al. 2021). Parallel experiments with the AFM force spectroscopy revealed that the bilayer stiffness in the presence of Aβ42 drops and the effect increases with the incubation time (Figure 2). Importantly, the stiffness of the bilayers over time approaches the values corresponding to the membrane stiffness without cholesterol. Edge‐height measurements of the bilayer at various time points revealed a progressive decrease in membrane thickness from the initial measurement up to 6 h. This reduction is consistent with a previous report (Kumarage et al. 2025) demonstrating a monotonic increase in membrane thickness with higher cholesterol content. Comparable trends in bilayer thinning and stiffness loss have been observed following treatment with the well‐established cholesterol‐extracting agent MβCD, supporting the interpretation that Aβ42 actively removes cholesterol from the membrane bilayer. This novel key finding on the ability of Aβ42 actively extracts cholesterol is directly demonstrated using a fluorescently labeled cholesterol analog (25‐NBD cholesterol). The fluorescence spectra of the solution above the bilayer reveal the accumulation of NBD cholesterol after its release from the bilayer (Figure 5). The cholesterol extraction is a time‐dependent process and the effects of cholesterol extraction are close to the one measured for MβCD. Although MβCD extracts cholesterol more rapidly, it was used at a concentration five orders of magnitude higher than Aβ42. After 6 h, Aβ42 achieved ~75% of the total MβCD‐mediated cholesterol release, highlighting its remarkable efficiency of Aβ42 regardless of a low concentration.

Computational modeling (López et al. 2013) suggests that MβCD facilitates cholesterol extraction through partial insertion into the membrane, allowing one of its rings to interact with the hydroxy group of the embedded cholesterol with an interaction distance of ~0.2 nm. In line with this model, recent MD simulations and in vitro fluorescence quenching experiments (Pinheiro et al. 2024) have shown that Aβ42 can partially insert into membranes, suggesting that a partially inserted Aβ42 may extract cholesterol. Neutron scattering experiments on POPC bilayer with cholesterol revealed the displacement of cholesterol towards the membrane surface in the presence of Aβ42 (Ashley et al. 2006), providing additional evidence to the extraction capability of Aβ42.

**FIGURE 7:**
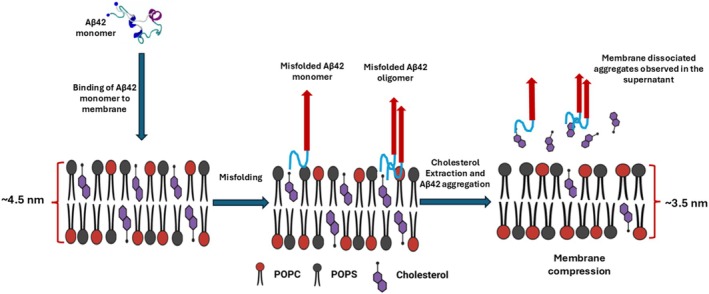
Aβ42 facilitates the extraction of cholesterol from the membrane at physiologically relevant concentrations. Membrane binding induces conformational misfolding of monomeric Aβ42, promoting its transition to a pathogenic state. The misfolded protein facilitates cholesterol extraction from the membrane while concurrently assembling into amyloid aggregates. Both the disruption of cholesterol homeostasis and the accumulation of Aβ aggregates synergistically exacerbate Alzheimer's disease pathology. [Figure created with BioRender.com].

Based on these findings, we propose the model for the Aβ42 interaction with membrane shown in Figure 7.

According to this model, Aβ42 after adsorption to the membrane surface and possible misfolding (step 1) extracts cholesterol from membrane (step 2). Extracted cholesterol accelerates aggregation of Aβ42 above the bilayer (step 3), which is in line with our recent publication (Hashemi et al. 2022), demonstrating that Aβ42 aggregation kinetics are significantly enhanced in the presence of free cholesterol. Computational modeling presented in the same study (Hashemi et al. 2022) reveals that the interaction of cholesterol with Aβ42 triggers a conformational switch, promoting its aggregation. Our model explains the presence of cholesterol within Aβ42 aggregates and, ultimately, in extracellular plaques, as reported previously (Mori et al. 2001; Panchal et al. 2010).

### 4.1 Cholesterol Extraction by Aβ42 and Potential Application to AD Development

To contextualize our findings, it is important to consider the physiological and pathological roles of cholesterol in the brain. Although the brain is rich in cholesterol, it does not appear in the free form. The major pool of cholesterol (70%–80%) is found in the myelin sheaths, with the rest of the lipids being in plasma membranes (Cao et al. 2024). Cholesterol is a highly functional molecule in the brain, so its concentrations are tightly regulated (Feringa and Van der Kant 2021). To this point, the dysregulation of cholesterol homeostasis is often associated with AD development, with its accumulation being one of the risk factors associated with disease development and progression (Sponne et al. 2004). For instance, in vivo studies (e.g., (Cantuti‐Castelvetri et al. 2018)) have shown that the intracellular accumulation of cholesterol in microglia induces maladaptive immune responses (such as cholesterol crystal formation, phagolysosomal membrane rupture, and stimulated inflammasomes), which impede remyelination and tissue regeneration. Cholesterol extraction by Aβ peptide contributes to this pathological effect of cholesterol.

Additionally, both in vitro and in vivo studies have shown that cholesterol accumulates within the mature senile amyloid plaques of AD (Mori et al. 2001; Panchal et al. 2010). Here, our findings on cholesterol extraction by Aβ42 offer a mechanistic explanation for the presence of cholesterol within Aβ42 aggregates and, ultimately, in amyloid plaques observed in AD brains, supporting our findings on the ability of Aβ42 to extract cholesterol from membranes to in vivo, resulting in the AD early onset and progression. These results support the view that cholesterol dysregulation contributes to AD pathology and may underlie previously observed histopathological features.

Depletion of cholesterol from the membrane leading to softening of membranes is another potentially pathological feature of Aβ42 associated with its interaction with membranes. Previous in vitro studies (Hering et al. 2003) have shown that depletion of membrane‐bound cholesterol results in the degeneration of dendritic spines and, ultimately, synapse loss—a common pathological feature observed in AD (Dorostkar et al. 2015). Cholesterol itself is largely a hydrophobic molecule primarily localized within the cell membranes, where it regulates membrane fluidity and can interact with neighboring lipids and proteins to regulate membrane trafficking or signal transduction (Luo et al. 2020). Cholesterol extraction decreases the membrane stiffness, which can result in deteriorating the function of membrane‐bound proteins. Specifically, lower cholesterol levels can inhibit APP (Amyloid Precursor Protein) processing by secretases (Feringa and Van der Kant 2021). Importantly, cholesterol molecules are not uniformly distributed within the cell membrane but, with other lipids, form cholesterol‐rich rafts. Their formation is critical for various membrane functions, including signal transduction (Liu et al. 2024). Aβ42‐mediated cholesterol depletion from these membrane domains can impair membrane dynamics and compromise the function of membrane proteins, many of which rely on the structural and mechanical properties of the lipid bilayer. Interestingly, β‐ and γ‐secretases are primarily localized in cholesterol‐enriched lipid rafts, so cholesterol depletion can alter their activity. Since these enzymes are responsible for APP cleavage and the production of Aβ peptides, including Aβ42, such changes can either increase or decrease Aβ levels (Grimm et al. 2013), both of which can contribute to AD pathology.

The association of cholesterol with Aβ42 aggregates is the factor that needs to be taken into account in the development of efficient anti‐amyloid immunotherapy (Yadollahikhales and Rojas 2023; Zhang et al. 2023). The presence of cholesterol in natural amyloid aggregates can be a factor contributing to the lack of their binding to antibodies raised to aggregates assembled without cholesterol.

In conclusion, the current view on the molecular mechanism of AD development is associated with the assembly of Aβ peptides in pathologic aggregates, Aβ oligomers specifically. We discovered a novel function of Aβ42 monomers, namely its capability to extract cholesterol from membranes. Interaction of Aβ42 with free cholesterol explains the molecular mechanism for the cholesterol dependent aggregation of Aβ42 on membranes. At the same time, the accumulation of free cholesterol and the increase of membrane fluidity after cholesterol depletion are the factors associated with AD‐related pathologies. Thus, additional Aβ42 associated pathologic factors were discovered, and this discovery changes the Aβ pathology paradigm. These findings suggest that therapeutic efforts for AD treatment should be focused on the development of efficient means of preventing or minimizing Aβ interaction with membranes. Moreover, given that the on‐membrane catalysis is the mechanism by which Aβ aggregation at physiologically relevant concentrations occurs (Lyubchenko 2023), the development of approaches affecting this pathology related interactions of Aβ should be the primary ones. Such approaches have the potential to pave the way for innovative combination therapies that could alleviate the suffering of millions of AD patients.

## Author Contributions

**Rishiram Baral:** investigation, methodology, validation, formal analysis, data curation, writing – original draft, visualization. **Ruan van Deventer:** methodology, validation, investigation, formal analysis, data curation, writing – original draft, visualization. **Yuri L. Lyubchenko:** conceptualization, supervision, funding acquisition, writing – review and editing, writing – original draft, project administration, methodology.

## Funding

This work was funded by the National Institutes of Health (NIH, GM100156) grant to Yuri L. Lyubchenko.

## Conflicts of Interest

The authors declare no conflicts of interest.

## Supporting information


**Figure S1:** AFM image illustrating the formation of a 0.25 mg/mL POPC:POPS supported lipid bilayer (SLB) incorporating cholesterol, with the corresponding edge height.
**Figure S2:** AFM time‐lapse images illustrating the aggregation of 50 nM Aβ42 on POPC:POPS bilayer with and without Cholesterol.
**Figure S3:** Quantitative analysis of Aβ42 aggregate volume on POPC:POPS bilayers in the presence and absence of cholesterol.
**Figure S4:** Histogram for values of Young's modulus of POPC:POPS phospholipid bilayer over 6‐h duration with different mol% of Cholesterol without Aβ42 (Control experiment).
**Figure S5:** Histogram for values of Young's modulus of POPC:POPS phospholipid bilayer over 6‐h duration with different mol% of Cholesterol in the presence of 50 nM Aβ42.
**Figure S6:** AFM images and thickness measurement profile on POPC:POPS with 20% Chol bilayer incubated with Aβ42 on top of the bilayer.
**Figure S7:** AFM images and thickness measurement profile on POPC:POPS with 20% Chol bilayer incubated with buffer (Control).
**Figure S8:** Histogram for values of Young's modulus of POPC:POPS: 20% Chol phospholipid bilayer over 6‐h duration in the presence of 50 nM Aβ42.
**Figure S9:** AFM images and thickness measurement profile on POPC:POPS bilayer incubated with 50 nM Aβ42.
**Figure S10:** AFM images and thickness measurement profile on POPC:POPS bilayer incubated with buffer (Control).
**Figure S11:** AFM images and thickness measurement profile on POPC:POPS with 20% Chol bilayer incubated with 3 mM MβCD.
**Figure S12:** AFM images and thickness measurement profile on POPC:POPS with 20% 25‐NBD Chol bilayer incubated with 50 nM Aβ42.
**Table S1:** Roughness (pm) of the bilayer without Aβ42.
**Table S2:** Young's Modulus (MPa) of the bilayer without Aβ42 (Experiment 1).
**Table S3:** Young's Modulus (MPa) of the bilayer without Aβ42 (Experiment 2).
**Table S4:** Young's Modulus (MPa) of the bilayer without Aβ42 (Experiment 3).
**Table S5:** Mean Young's modulus (MPa) of the bilayer, calculated from three independent experiments without Aβ42.
**Table S6:**Young's Modulus (MPa) of the bilayer with Aβ42 (Experiment 1).
**Table S7:** Young's Modulus (MPa) of the bilayer with Aβ42 (Experiment 2).
**Table S8:** Young's Modulus (MPa) of the bilayer with Aβ42 (Experiment 3).
**Table S9:** Mean Young's modulus (MPa) of the bilayer, calculated from three independent experiments with Aβ42.
**Table S10:** Thickness (nm) of the bilayer with 20% Cholesterol in the presence of Aβ42.
**Table S11:** Thickness (nm) of the bilayer with 20% Cholesterol in the presence of buffer (Control).
**Table S12:** Young's modulus (MPa) of the bilayer with 20% Cholesterol in the presence of Aβ42.
**Table S13:** Young's modulus (MPa) of the bilayer containing 20% cholesterol in the absence of Aβ42.
**Table S14:** Thickness (nm) of the POPC/POPS only bilayer in the presence of Aβ42.
**Table S15:** Thickness (nm) of the POPC/POPS only bilayer in the presence of buffer (Control).
**Table S16:** Young's modulus (MPa) of the bilayer with 20% Cholesterol in the presence of MβCD.
**Table S17:** Thickness (nm) of the bilayer with 20% Cholesterol in the presence of MβCD.
**Table S18:** Maxima values of fluorescence intensity (a.u.) recorded at each time point for PC:PS 20% NBD Chol bilayer incubated with 50 nM Aβ42.
**Table S19:** Maxima values of fluorescence intensity (a.u.) recorded at each time point for PC:PS 20% NBD Chol bilayer incubated with 3 mM MβCD.
**Table S20:** Maxima values of fluorescence intensity (a.u) recorded at each time point for PC:PS 20% NBD Chol bilayer incubated with 10 mM HEPES (control experiment).
**Table S21:** Young's modulus (MPa) of the bilayer with 20% 25‐NBD Cholesterol in the presence of Aβ42.
**Table S22:** Thickness (nm) of the bilayer with 25‐NBD cholesterol in the presence of 50 nM Aβ42.

## Data Availability

The data that support the findings of this study are openly available upon request to the authors and in Supporting Information. The preprint of this article was posted on bioRxiv; 24/08/2025; https://doi.org/10.1101/2025.08.20.671368.
