# Supplementary material for: Amyloid β—Cholesterol Interplay: Removal of Cholesterol From the Membranes to Catalyze Aggregation and Amyloid Pathology
Source: J Neurochem. 2026 Feb 10;170(2):e70380. doi: 10.1111/jnc.70380 (PMC13007493; doi:10.1111/jnc.70380)
Supplement: Supplementary file 1 — Figure S1: AFM image illustrating the formation of a 0.25 mg/mL POPC:POPS supported lipid bilayer (SLB) incorporating cholesterol, with the corresponding edge height. Figure S2: AFM time‐lapse images illustrating the aggregation of 50 nM Aβ42 on POPC:POPS bilayer with and without Cholesterol. Figure S3: Quantitative analysis of Aβ42 aggregate volume on POPC:POPS bilayers in the presence and absence of cholesterol. Figure S4: Histogram for values of Young's modulus of POPC:POPS phospholipid bilayer over 6‐h duration with different mol% of Cholesterol without Aβ42 (Control experiment). Figure S5: Histogram for values of Young's modulus of POPC:POPS phospholipid bilayer over 6‐h duration with different mol% of Cholesterol in the presence of 50 nM Aβ42. Figure S6: AFM images and thickness measurement profile on POPC:POPS with 20% Chol bilayer incubated with Aβ42 on top of the bilayer. Figure S7: AFM images and thickness measurement profile on POPC:POPS with 20% Chol bilayer incubated with buffer (Control). Figure S8: Histogram for values of Young's modulus of POPC:POPS: 20% Chol phospholipid bilayer over 6‐h duration in the presence of 50 nM Aβ42. Figure S9: AFM images and thickness measurement profile on POPC:POPS bilayer incubated with 50 nM Aβ42. Figure S10: AFM images and thickness measurement profile on POPC:POPS bilayer incubated with buffer (Control). Figure S11: AFM images and thickness measurement profile on POPC:POPS with 20% Chol bilayer incubated with 3 mM MβCD. Figure S12: AFM images and thickness measurement profile on POPC:POPS with 20% 25‐NBD Chol bilayer incubated with 50 nM Aβ42. Table S1: Roughness (pm) of the bilayer without Aβ42. Table S2: Young's Modulus (MPa) of the bilayer without Aβ42 (Experiment 1). Table S3: Young's Modulus (MPa) of the bilayer without Aβ42 (Experiment 2). Table S4: Young's Modulus (MPa) of the bilayer without Aβ42 (Experiment 3). Table S5: Mean Young's modulus (MPa) of the bilayer, calculated from three independent experiment [file JNC-170-e70380-s001.pdf]

Supporting Information

Amyloid β - cholesterol interplay: Removal of cholesterol from the membranes to catalyze aggregation and amyloid pathology

Rishiram Baral[1], Ruan van Deventer[1] and Yuri L. Lyubchenko[1]*

[1]Department of Pharmaceutical Sciences, University of Nebraska Medical Center, Omaha, NE 68198-6025, USA

* Corresponding author

Email: ylyubchenko@unmc.edu

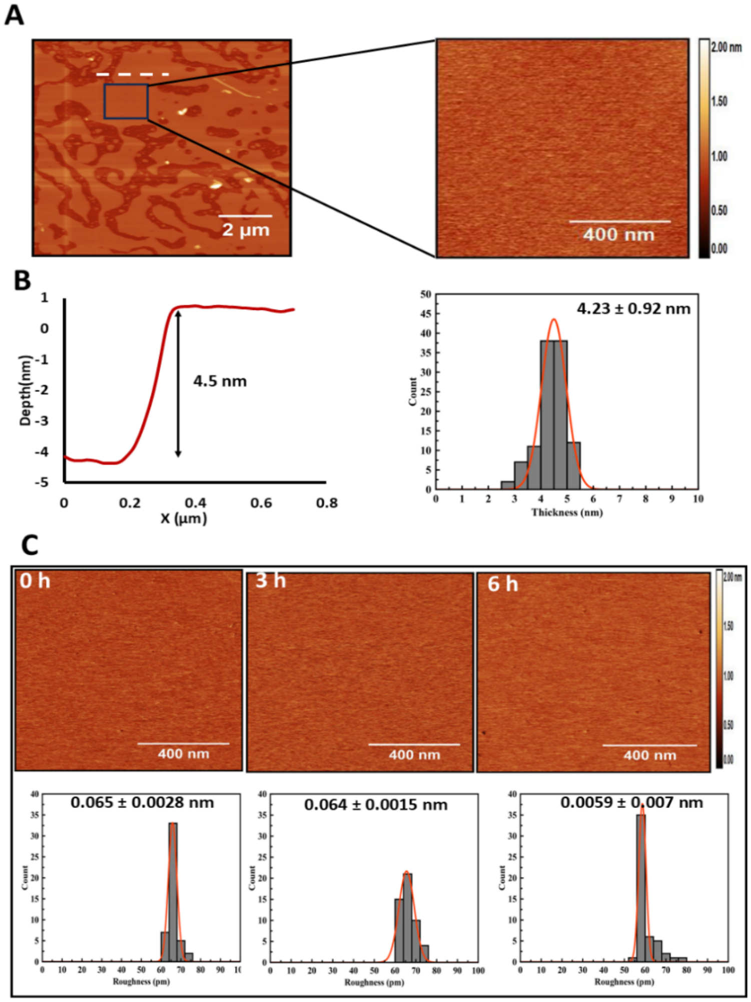

Figure S1: AFM image illustrating the formation of a 0.25 mg/mL POPC:POPS supported lipid bilayer (SLB) incorporating cholesterol, with the corresponding edge height. (A) AFM image of size 10 x 10 µm scan of POPC:POPS:20% Chol SLB showing appropriate surface coverage as well as edge. Few white features present on the surface are the unruptured vesicles of cholesterol. the zoomed area shows the AFM image of 1 x 1 µm scan of the smooth surface of the SLB. In all the assembled SLB with different mol% of cholesterol, similar surface of 1 x 1 µm was selected for time-lapse AFM imaging until 6 hours. (B) Height of the edge of the SLB which was measured as 4.5 nm. The cross section was performed on the edge which is shown with white dashed line in Figure S1A. Several cross-sections was performed on the edge of the bilayer (n=100). The histograms were approximated with Gaussians. The mean height obtained from the approximations along with the standard deviations is shown on right hand side. (C) Time-lapse AFM images with corresponding RMS roughness of 0.25 mg/mL POPC:POPS:20% Chol bilayer (n=50). The measurement was performed at 0 h, 3 h and 6 h of 1 x 1 µm smooth area of bilayer. The histograms were approximated with Gaussians. The mean RMS roughness obtained from the approximations along with the standard deviations are shown inside each frame.

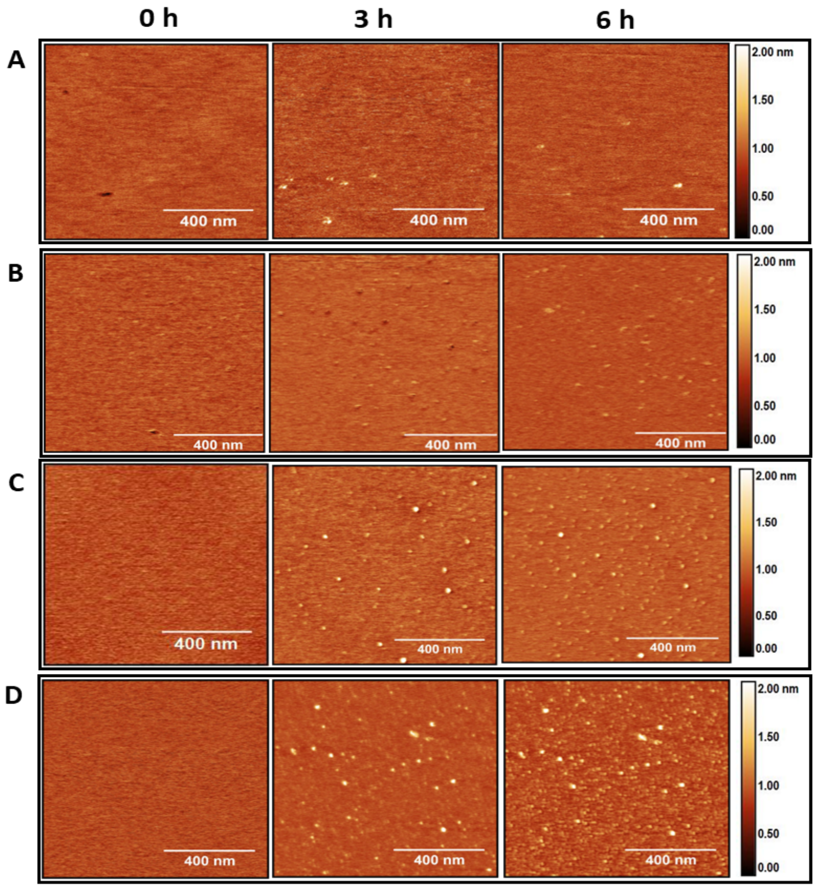

Figure S2: AFM time-lapse images illustrating the aggregation of 50 nM Aβ42 on POPC:POPS bilayer with and without Cholesterol. Aggregation of 50 nM Aβ42 on 0.25mg/mL POPC:POPS lipid bilayer until 6 hour; (A) without cholesterol (B) with 10% cholesterol (C) with 20% cholesterol and (D) with 30% cholesterol. The scan size is 1 x 1 µm. The number and sizes of the aggregates exhibit a progressive increase over time, with the cholesterol concentration being the highest for the bilayer with 30% cholesterol.

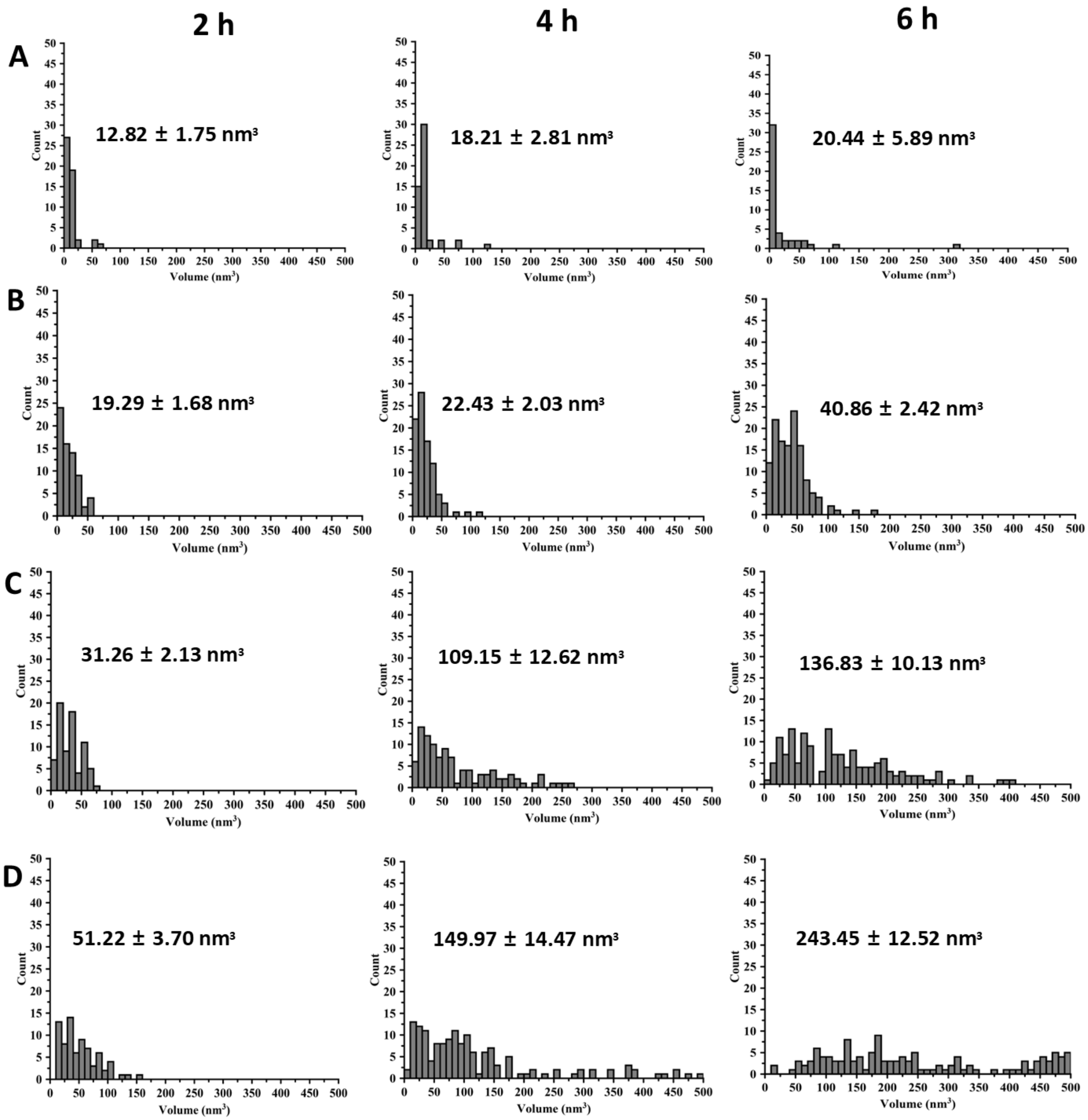

Figure S3:Quantitative analysis of Aβ42 aggregate volume on POPC:POPS bilayers in the presence and absence of cholesterol. The volume of Aβ42 aggregates was measured on 0.25 mg/mL POPC:POPS lipid bilayers over a 6-hour period under four conditions: (A) without cholesterol, (B) with 10% cholesterol, (C) with 20% cholesterol, and (D) with 30% cholesterol. Data are presented as mean ± mean absolute difference (MAD). A time-dependent increase in aggregate volume was observed in all conditions, with the highest accumulation detected on bilayers containing 30% cholesterol.

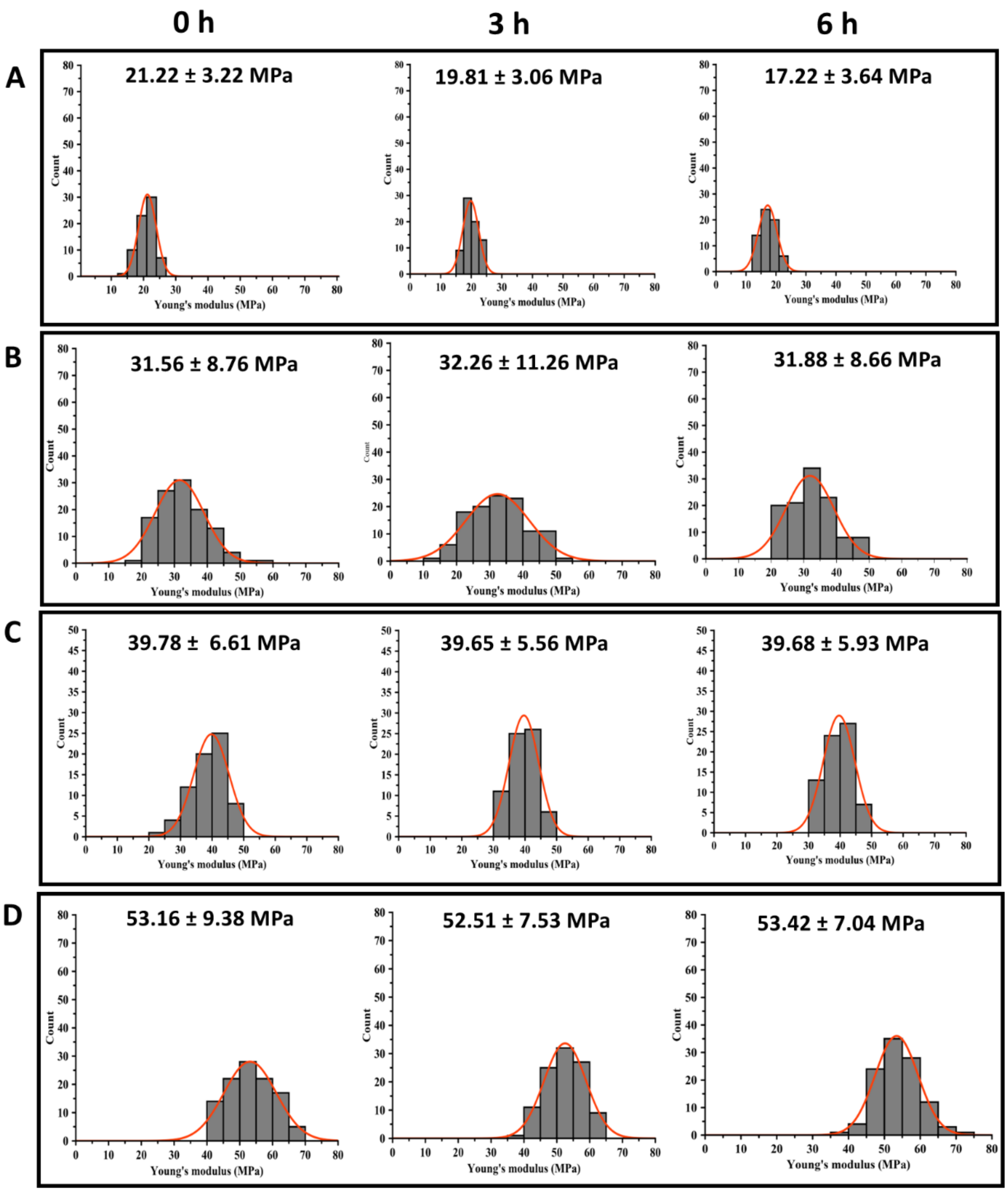

Figure S4: Histogram for values of Young's modulus of POPC:POPS phospholipid bilayer over 6-hour duration with different mol% of Cholesterol without Aβ42 (Control experiment). The Young's modulus of 0.25 mg/mL POPC:POPS bilayer with (A) POPC:POPS, (B) POPC:POPS:10% Chol, (C) POPC:POPS:20% Chol, (D) POPC:POPS:30% Chol from 0 time point to 6 h in the presence of 10 mM HEPES buffer with salt (n=100) was measured. The modulus data obtained from each force points of the scanned surface were approximated with Gaussians. The mean values obtained from the approximations along with the standard deviations are shown inside each frame.

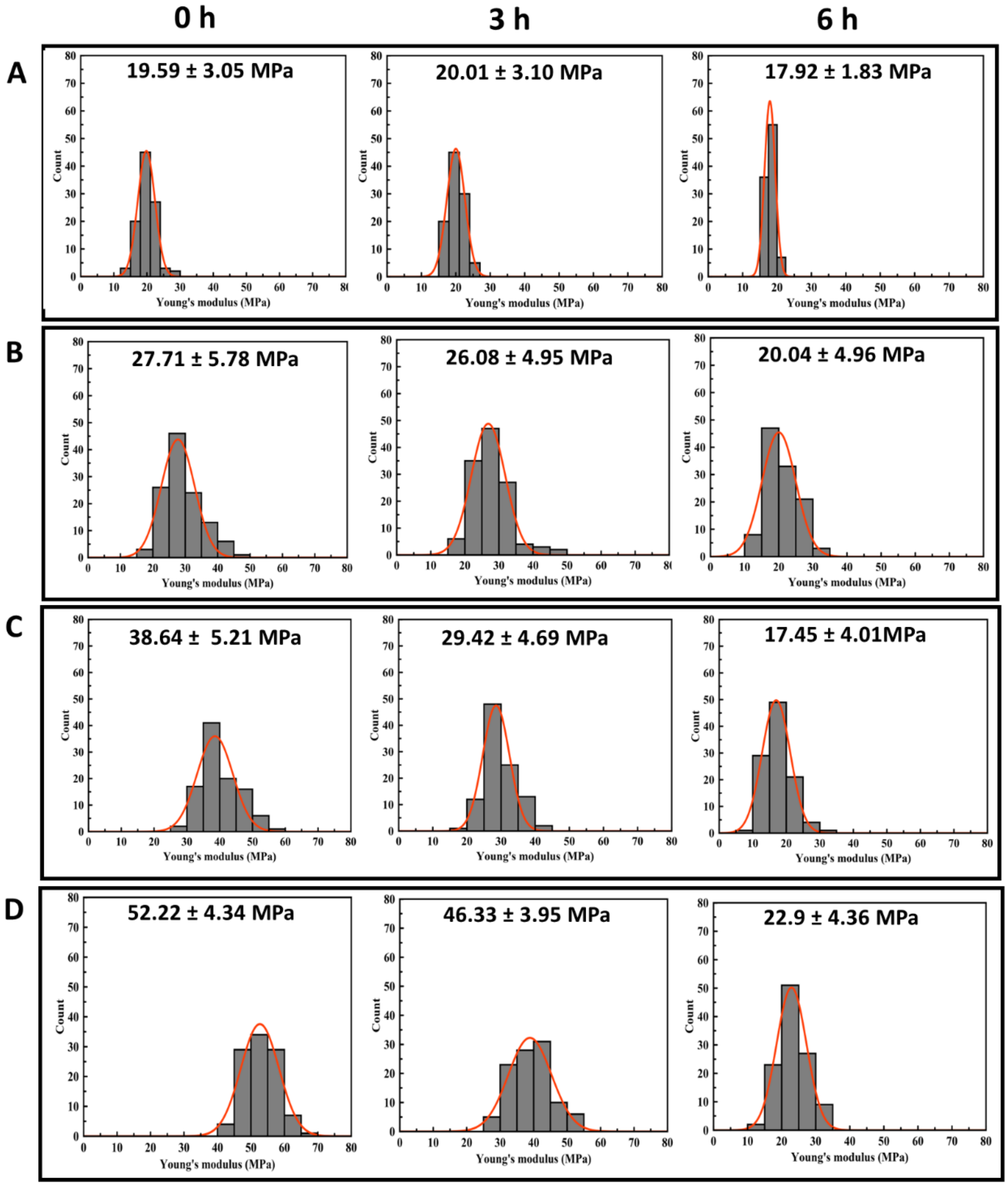

Figure S5: Histogram for values of Young's modulus of POPC:POPS phospholipid bilayer over 6-hour duration with different mol% of Cholesterol in the presence of 50 nM Aβ42. The Young's modulus of 0.25 mg/mL POPC:POPS bilayer with (A) POPC:POPS, (B) POPC:POPS:10% Chol, (C) POPC:POPS:20% Chol, (D) POPC:POPS:30% Chol from 0 time point to 6 h in the presence of 50 nM Aβ42 (n=100) was measured. The modulus data obtained from each force points of the scanned surface were approximated with Gaussians. The mean values obtained from the approximations along with the standard deviations are shown inside each frame.

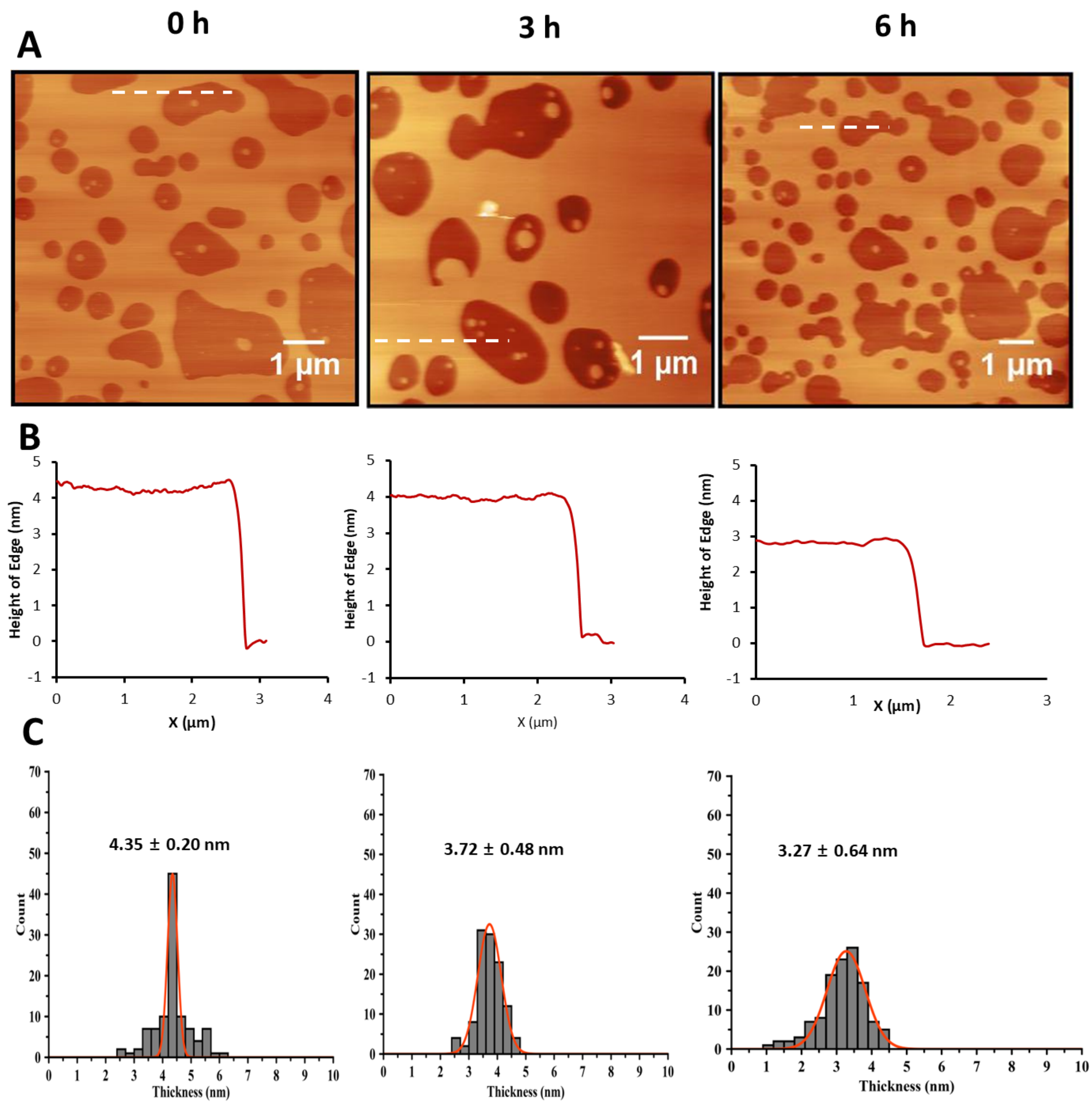

Figure S6: AFM images and thickness measurement profile on POPC:POPS with 20% Chol bilayer incubated with Aβ42 on top of the bilayer. (A) Representative AFM image of 0.25 mg/mL POPC:POPS:20% Chol phospholipid bilayer incubated with Aβ42 where the thickness was measured. The scan size is 7 x 7 µm (B) The representative height traces correspond to the horizontal cross section shown by white line in Figure S6A. (C) The thickness of the bilayer with horizontal cross-section was measured at various locations near to the edge. The histograms were approximated with Gaussians. The mean thickness values obtained from the approximations along with the standard deviation are shown inside each frame.

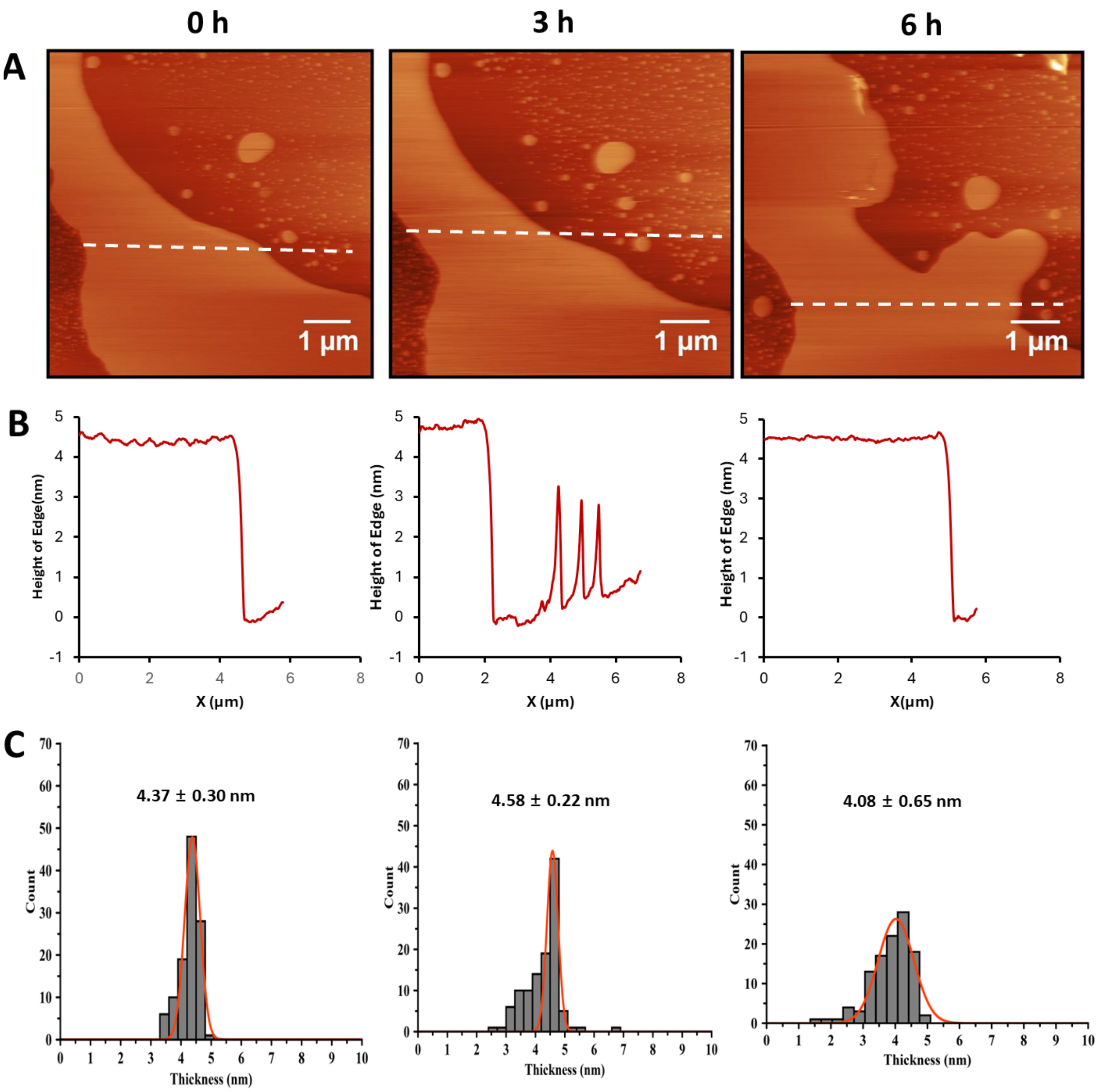

Figure S7: AFM images and thickness measurement profile on POPC:POPS with 20% Chol bilayer incubated with buffer (Control). (A) Representative AFM image of 0.25 mg/mL POPC:POPS:20% Chol phospholipid bilayer incubated with buffer where the thickness was measured. The scan size is 7 x 7 µm (B) The representative height traces correspond to the horizontal cross section shown by white line in Figure S7A. (C) The thickness of the bilayer with horizontal cross-section was measured at various locations near to the edge. The histograms were approximated with Gaussians. The mean thickness values obtained from the approximations along with the standard deviation are shown inside each frame.

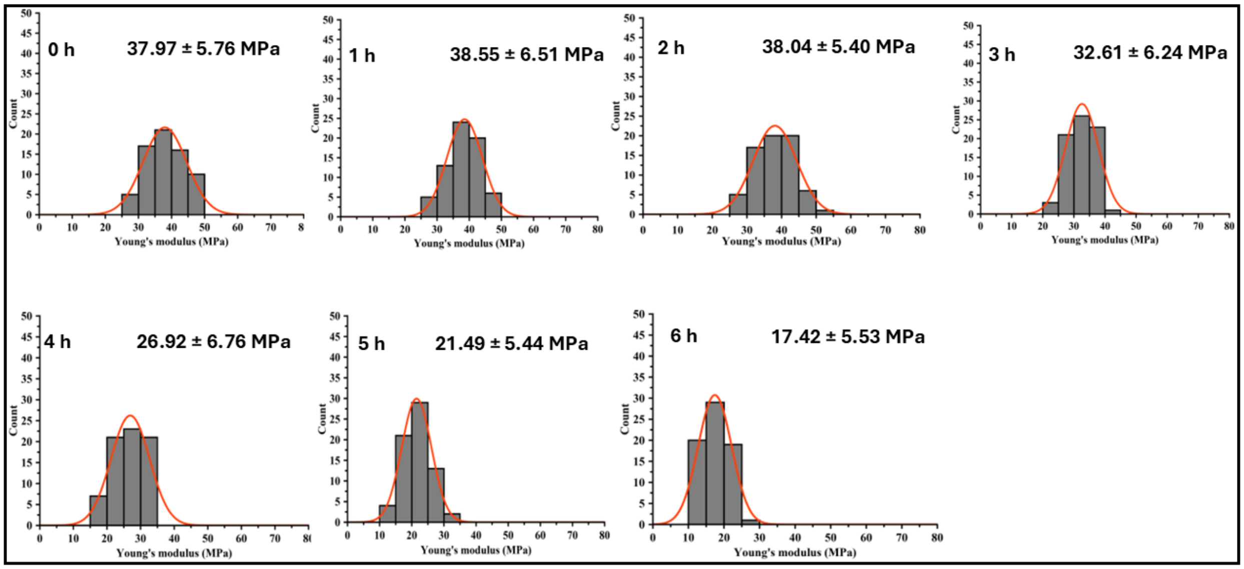

Figure S8: Histogram for values of Young's modulus of POPC:POPS: 20% Chol phospholipid bilayer over 6-hour duration in the presence of 50 nM Aβ42. The Young's modulus of 0.25 mg/mL POPC:POPS:20% Chol from 0 time point to 6 h in the presence of 50 nM Aβ42 (n=100) was measured. The modulus data obtained from each force points of the scanned surface were approximated with Gaussians. The mean values obtained from the approximations along with the standard deviations are shown inside each frame.

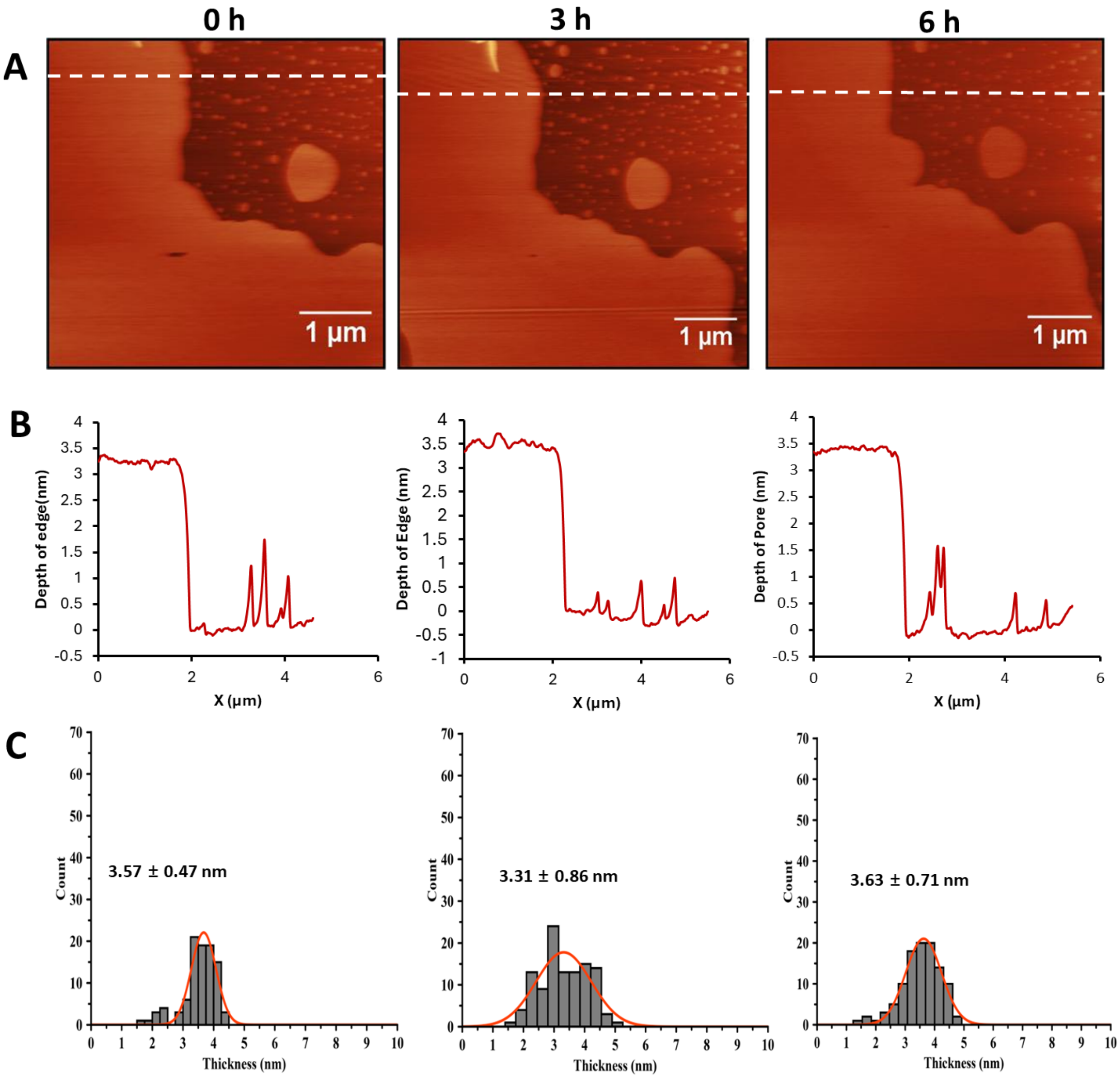

Figure S9: AFM images and thickness measurement profile on POPC:POPS bilayer incubated with 50 nM Aβ42. (A) Representative AFM image of 0.25 mg/mL POPC:POPS phospholipid bilayer incubated with buffer where the thickness was measured. The scan size is 7 x 7 µm (B) The representative height traces correspond to the horizontal cross section shown by white line in Figure S9A. (C) The thickness of the bilayer with horizontal cross-section was measured at various locations near to the edge. The histograms were approximated with Gaussians. The mean thickness values obtained from the approximations along with the standard deviation are shown inside each frame.

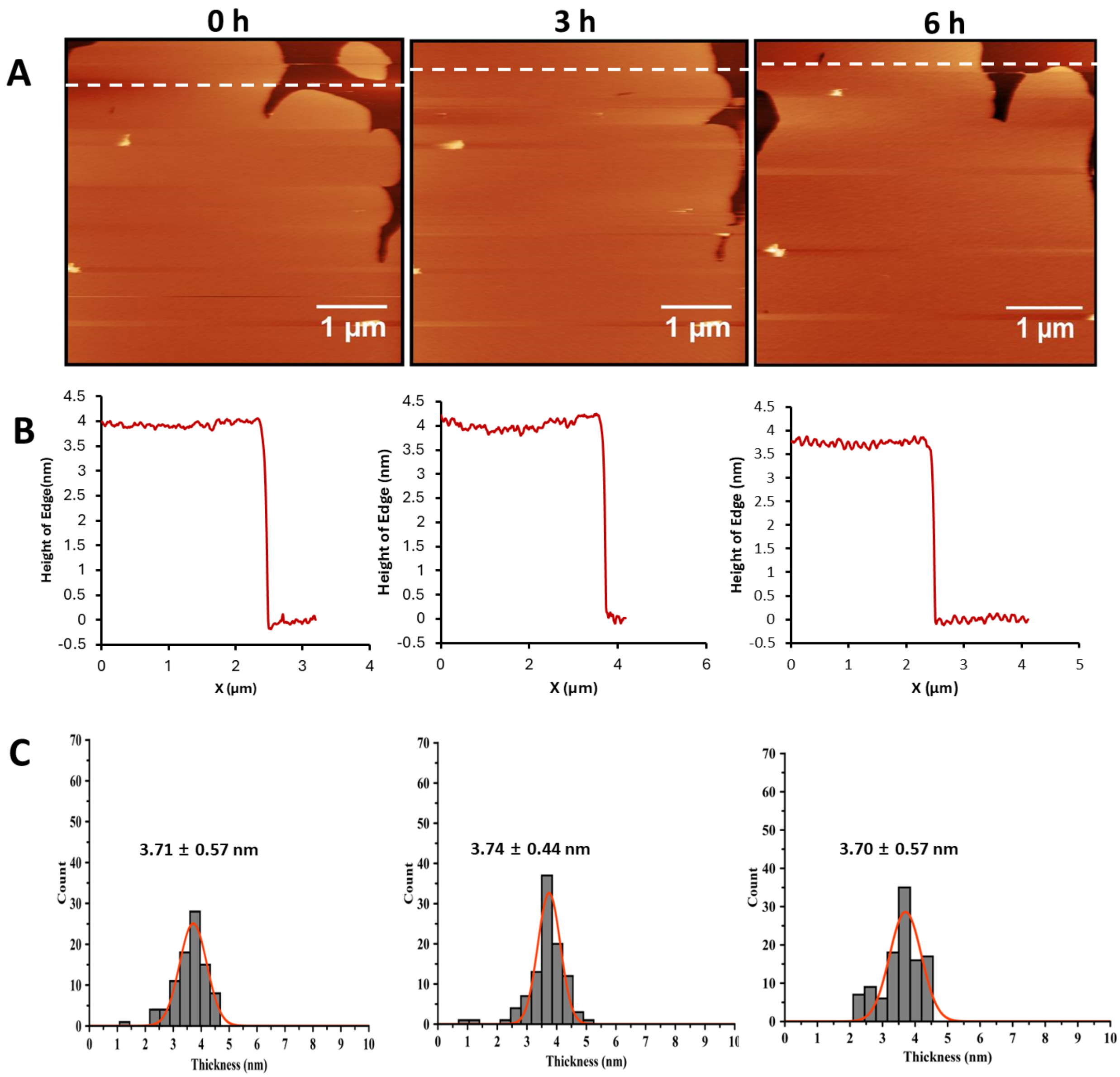

Figure S10. AFM images and thickness measurement profile on POPC:POPS bilayer incubated with buffer (Control). (A) Representative AFM image of 0.25 mg/mL POPC:POPS phospholipid bilayer incubated with buffer where the thickness was measured. The scan size is 7 x 7 μm (B) The representative height traces correspond to the horizontal cross section shown by white line in Figure S10A. (C) The thickness of the bilayer with horizontal cross-section was measured at various locations near to the edge. The histograms were approximated with Gaussians. The mean thickness values obtained from the approximations along with the standard deviation are shown inside each frame.

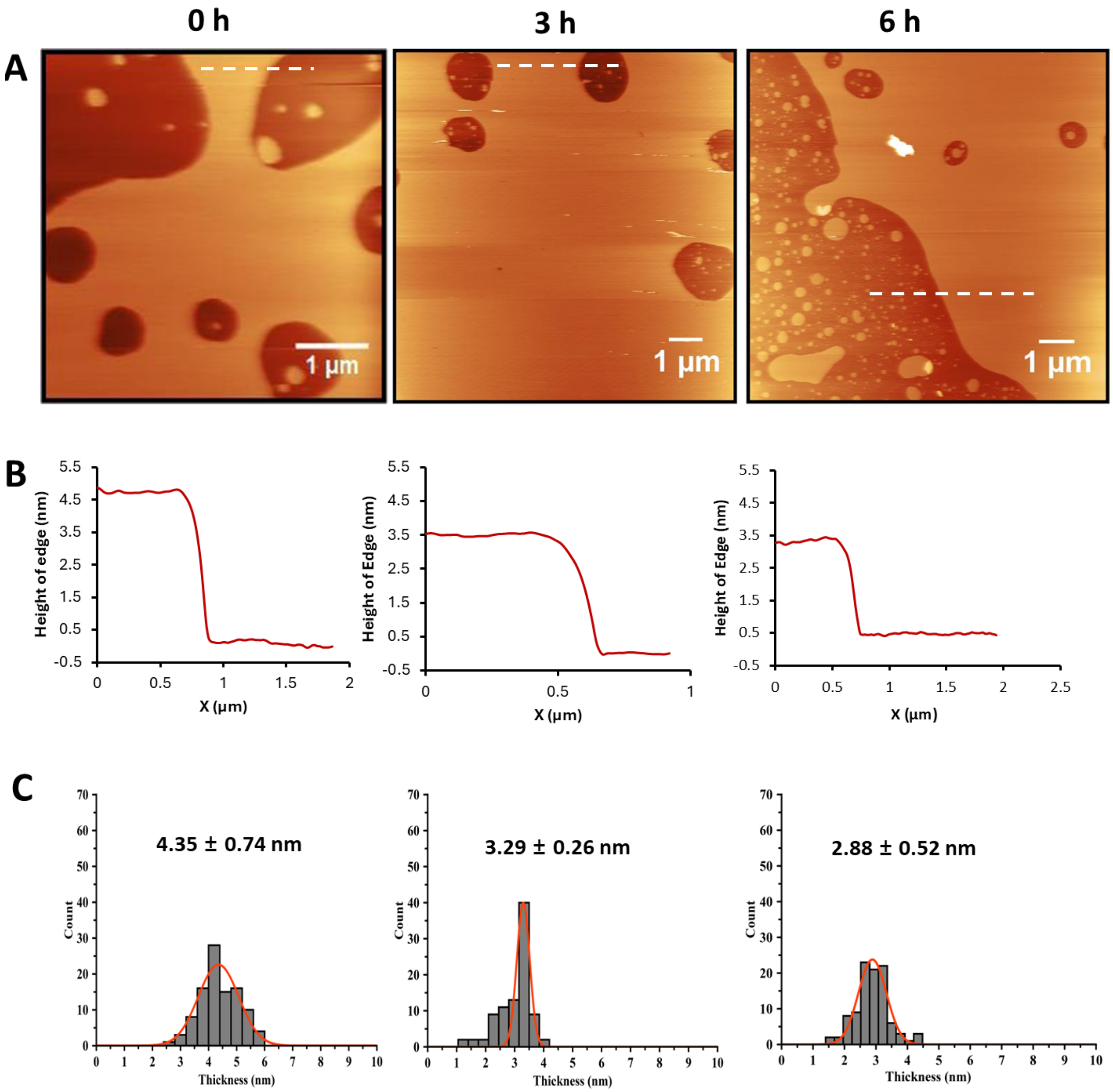

Figure S11. AFM images and thickness measurement profile on POPC:POPS with 20% Chol bilayer incubated with 3mM MβCD. (A) Representative AFM image of 0.25 mg/mL POPC:POPS:20% Chol phospholipid bilayer incubated with 3mM MβCD where the thickness was measured. The scan size is 7 x 7 µm (B) The representative height traces correspond to the horizontal cross section shown by white line in Figure S11A. (C) The thickness of the bilayer with horizontal cross-section was measured at various locations near to the edge. The histograms were approximated with Gaussians. The mean thickness values obtained from the approximations along with the standard deviation are shown inside each frame.

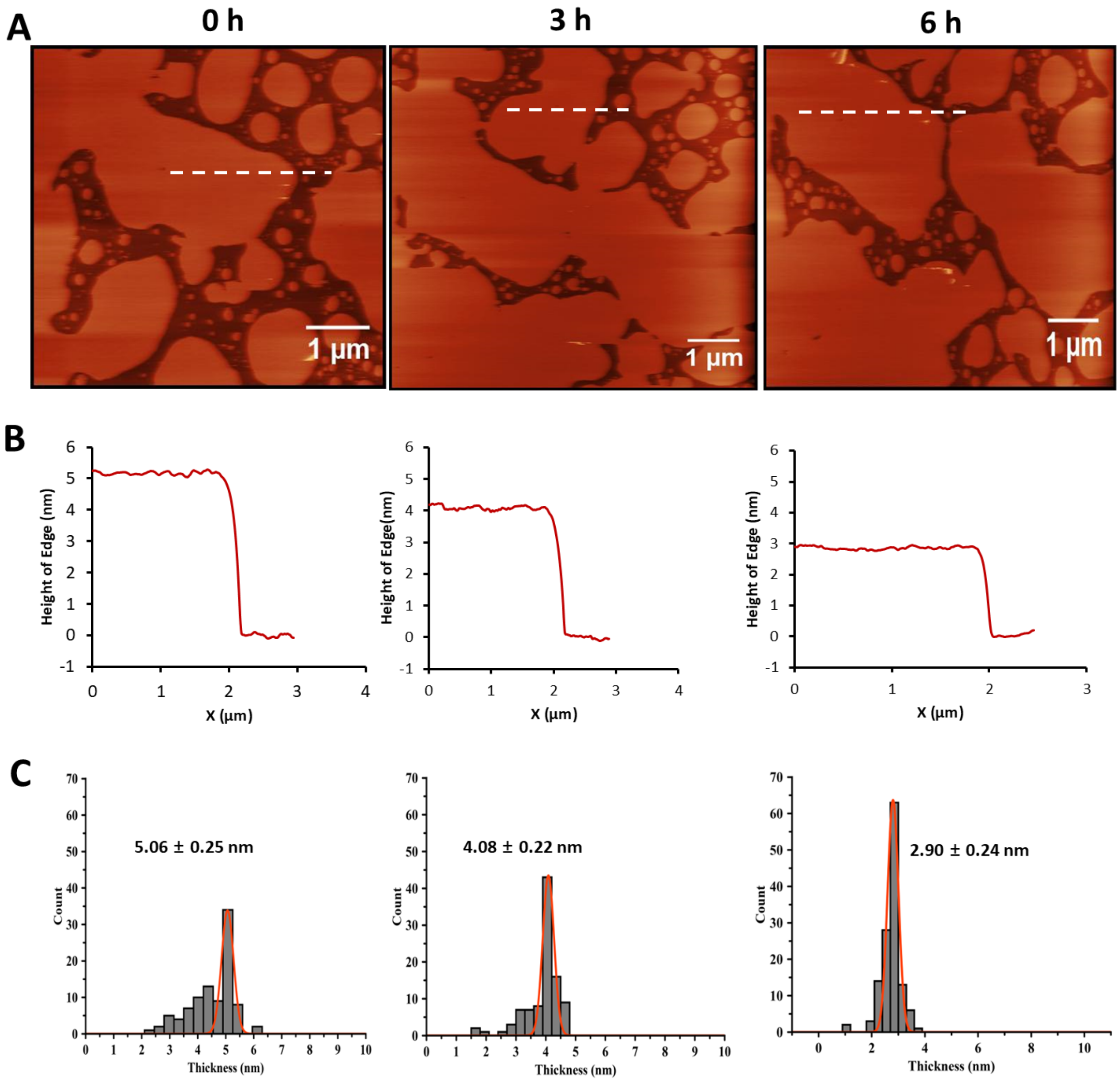

Figure S12. AFM images and thickness measurement profile on POPC:POPS with 20% 25-NBD Chol bilayer incubated with 50 nM Aβ42. (A) Representative AFM image of 0.25 mg/mL POPC:POPS:20% 25-NBD Chol phospholipid bilayer incubated with 50 nM Aβ42 where the thickness was measured. The scan size is 7 x 7 µm. (B) The representative height traces correspond to the horizontal cross section shown by white line in Figure S12A. (C) The thickness of the bilayer with horizontal cross-section was measured at various locations near to the edge. The histograms were approximated with Gaussians. The mean thickness values obtained from the approximations along with the standard deviation are shown inside each frame.

Supplementary Tables

Table S1. Roughness (pm) of the bilayer without Aβ42

Time (h)	PC:PS	PC:PS:10% Chol	PC:PS:20% Chol	PC:PS:30% Chol
**0**	59.47 ± 1.54	68.86 ± 4.32	67.41 ± 3.56	79.16 ± 2.54
**1**	58.44 ± 0.86	67.33 ± 3.11	67.65 ± 2.30	78.16 ± 1.21
**2**	59.04 ± 0.42	72.44 ± 8.44	69.42 ± 3.21	81.82 ± 1.01
**3**	59.33 ± 0.94	66.52 ± 4.44	73.42 ± 5.72	76.52 ± 2.02
**4**	60.09 ± 0.43	68.76 ± 1.95	74.7 ± 2.28	73.44 ± 1.51
**5**	60.08 ± 0.54	69.91 ± 6.97	71.24 ± 6.10	96.12 ± 1.17
**6**	62 ± 1.12	70.92 ± 7.42	71.36 ± 2.99	90.68 ± 3.02

Data are expressed as Mean± mean absolute difference (MAD) (n=10)

Table S2. Young's Modulus (MPa) of the bilayer without Aβ42 (Experiment 1)

Time (h)	PC:PS	PC:PS:10% Chol	PC:PS:20% Chol	PC:PS:30% Chol
**0**	21.83 ± 6.71	32.06 ± 5.29	43.42 ± 5.98	49.28 ± 5.81
**1**	21.17 ± 6.12	32.26 ± 5.36	42.61 ± 4.69	49.36 ± 7.57
**2**	19.15 ± 5.01	28.92 ± 6.83	41.54 ± 5.56	50.21± 6.61
**3**	18.91 ± 6.05	30.57 ± 5.08	41.73 ± 7.65	48.70 ± 5.99
**4**	20.45 ± 4.58	30.42 ± 6.53	44.61 ± 6.96	48.86 ± 5.90
**5**	18.18 ± 4.37	31.98 ± 6.95	43.47 ± 7.55	49.97 ± 6.42
**6**	20.45 ± 4.78	31.57 ± 5.36	44.99 ± 5.28	48.70 ± 5.55

Data are expressed as Mean± SD (n=100)

Table S3. Young's Modulus (MPa) of the bilayer without Aβ42 (Experiment 2)

Time (h)	PC:PS	PC:PS:10% Chol	PC:PS:20% Chol	PC:PS:30% Chol
**0**	21.22 ± 3.22	31.56 ± 8.76	39.78 ± 6.61	53.16 ± 9.38
**1**	19.61 ± 5.86	31.54 ± 5.94	39.45 ± 6.17	53.17 ± 8.14
**2**	17.73 ± 4.8	28.27 ± 4.80	38.47 ± 5.22	53.59 ± 7.11
**3**	19.81 ± 3.06	32.26 ± 11.26	39.65 ± 5.56	52.51 ± 7.53
**4**	18.94 ± 4.39	29.74 ± 4.39	41.31 ± 8.51	54.46 ± 6.35
**5**	16.34 ± 4.19	29.08 ± 4.19	40.25 ± 7.74	52.50 ± 6.91
**6**	17.22 ± 3.64	31.88 ± 8.66	39.68 ± 5.93	53.42 ± 7.04

Data are expressed as Mean± SD (n=100)

Table S4. Young's Modulus (MPa) of the bilayer without Aβ42 (Experiment 3)

Time (h)	PC:PS	PC:PS:10% Chol	PC:PS:20% Chol	PC:PS:30% Chol
**0**	19.28 ± 5.45	30.29 ± 5.02	36.18 ± 4.91	47.80 ± 1.72
**1**	17.27 ± 8.92	33.45 ± 5.09	35.50 ± 4.98	49.75 ± 1.60
**2**	21.24 ± 6.78	29.28 ± 4.82	34.62 ± 6.35	48.70± 1.83
**3**	20.24 ± 5.87	28.94 ± 6.60	34.77 ± 4.72	50.38 ± 2.32
**4**	18.34 ± 6.93	30.45 ± 5.09	37.17 ± 4.98	48.70 ± 2.52
**5**	22.36 ± 3.48	32.56 ± 6.20	36.22 ± 6.98	47.86 ± 1.59
**6**	18.86 ± 4.39	29.89 ± 4.82	37.49 ± 7.32	48.70 ± 2.09

Data are expressed as Mean± SD (n=100)

Table S5. Mean Young's modulus (MPa) of the bilayer, calculated from three independent experiments without Aβ42

Time (h)	PC:PS	PC:PS:10% Chol	PC:PS:20% Chol	PC:PS:30% Chol
**0**	20.44 ± 0.92	31.23 ± 0.62	39.93 ± 2.50	49.83 ± 1.72
**1**	19.35 ± 1.38	32.41 ± 0.68	39.18 ± 2.45	50.76 ± 1.60
**2**	19.37 ± 1.24	28.92 ± 0.23	38.21 ± 2.39	50.83 ± 1.83
**3**	18.88 ± 0.91	29.8 ± 0.57	38.38 ± 2.40	50.53 ± 1.32
**4**	19.24 ± 0.80	30.20 ± 0.30	41.03 ± 2.57	50.67 ± 2.52
**5**	18.96 ± 2.26	31.20 ± 1.41	39.98 ± 2.50	50.11 ± 1.59
**6**	19.41 ± 0.68	30.05 ± 1.01	41.38 ± 2.59	50.27 ± 2.09

Data are expressed as mean ± MAD

Table S6. Young's Modulus (MPa) of the bilayer with Aβ42 (Experiment 1)

Time (h)	PC:PS	PC:PS:10% Chol	PC:PS:20% Chol	PC:PS:30% Chol
**0**	20.12 ± 4.84	27.84 ± 5.78	38.64 ± 5.21	51.14 ± 4.34
**1**	19.59 ± 6.37	27.71 ± 4.99	37.49 ± 5.27	52.22 ± 6.13
**2**	18.97 ± 3.15	28.42 ± 4.32	37.38 ± 5.63	52.68 ± 5.75
**3**	19.18 ± 3.99	26.08 ± 4.95	29.42 ± 4.69	46.33 ± 3.95
**4**	18.87 ± 3.47	20.47 ± 4.32	27.04 ± 3.79	38.88 ± 6.48
**5**	17.77 ± 3.67	21.36 ± 6.58	18.31 ± 3.69	28.49 ± 3.66
**6**	17.74 ± 3.82	20.04 ± 4.96	17.45 ± 4.01	22.9 ± 4.36

Data are expressed as Mean± SD (n=100)

Table S7. Young's Modulus (MPa) of the bilayer with Aβ42 (Experiment 2)

Time (h)	PC:PS	PC:PS:10% Chol	PC:PS:20% Chol	PC:PS:30% Chol
**0**	20.69 ± 3.35	32.25 ± 6.23	38.89 ± 6.7	53.13 ± 4.90
**1**	20.05 ± 3.71	31.22 ± 5.44	37.67 ± 6.2	50.57 ± 4.65
**2**	19.75 ± 4.21	28.99 ± 7.05	36.77 ± 8.1	48.18 ± 3.16
**3**	20.33 ± 3.72	23.57 ± 5.06	27.07 ± 4.4	40.43 ± 5.18
**4**	17.52 ± 3.75	21.01 ± 5.18	24.05 ± 6.3	37.32 ± 5.92
**5**	17.72 ± 2.41	17.98± 4.06	25.06 ± 5.4	27.35 ± 3.48
**6**	17.43 ± 2.72	16.96 ± 4.34	18.37 ± 6.5	21.98 ± 6.89

Data are expressed as Mean± SD (n=100)

Table S8. Young's Modulus (MPa) of the bilayer with Aβ42 (Experiment 3)

Time (h)	PC:PS	PC:PS:10% Chol	PC:PS:20% Chol	PC:PS:30% Chol
**0**	19.9 ± 3.28	27.79 ± 4.34	40.72 ± 9.04	51.05 ± 6.74
**1**	20.21 ± 2.42	28.12 ± 4.05	38.56 ± 10.34	51.49 ± 6.32
**2**	20.37 ± 2.7	28.1 ± 3.92	36.41 ± 7.42	45.29 ± 4.34
**3**	18.87 ± 2.45	22.68 ± 3.02	31.98 ± 4.55	38.00 ± 7.12
**4**	18.24 ± 1.82	19.73 ± 2.94	28.65 ± 4.57	27.85 ± 4.02
**5**	18.32 ± 1.41	17.38± 3.15	21.31 ± 4.81	22.38 ± 4.79
**6**	17.55 ± 1.65	16.15 ± 3.92	17.86 ± 3.72	18.38 ± 5.65

Data are expressed as Mean± SD (n=100)

Table S9. Mean Young's modulus (MPa) of the bilayer, calculated from three independent experiments with Aβ42

Time (h)	PC:PS	PC:PS:10% Chol	PC:PS:20% Chol	PC:PS:30% Chol
**0**	20.23 ± 0.30	29.29 ± 1.97	39.41 ± 0.86	51.77 ± 0.90
**1**	19.95 ± 0.24	29.01 ± 1.46	37.90 ± 0.43	51.42 ± 0.57
**2**	19.69 ± 0.48	28.63 ± 0.35	36.85 ± 0.35	48.71 ± 2.64
**3**	19.46 ± 0.58	24.11 ± 1.31	29.49 ± 1.66	41.58 ± 3.16
**4**	18.21 ± 0.46	20.40 ± 0.44	26.58 ± 1.68	34.68 ± 4.55
**5**	17.93 ± 0.26	18.90 ± 0.63	21.56 ± 2.33	26.20 ± 2.55
**6**	17.557 ± 0.11	17.71 ± 1.54	17.89 ± 0.31	21.08 ± 1.80

Data are expressed as Mean ± MAD

Table S10. Thickness (nm) of the bilayer with 20% Cholesterol in the presence of Aβ42

Time (h)	Experiment 1	Experiment 2	Mean ± MAD
**0**	4.35 ± 0.20	4.30 ± 0.58	4.50 ± 0.025
**3**	3.72 ± 0.48	3.60 ± 0.46	3.55 ± 0.06
**6**	3.27 ± 0.64	3.26 ± 0.78	3.00 ± 0.005

Data from individual experiments are reported as Mean ± SD, whereas the pooled results from three experiments are presented as Mean ± MAD

Table S11. Thickness (nm) of the bilayer with 20% Cholesterol in the presence of buffer (Control)

Time (h)	Experiment 1	Experiment 2	Mean ± MAD
**0**	4.37 ± 0.30	4.42 ± 0.51	4.39 ± 0.025
**3**	4.58 ± 0.22	4.76 ± 0.35	4.67 ± 0.09
**6**	4.08 ± 0.65	4.37 ± 0.36	4.22 ± 0.15

Data from individual experiments are reported as Mean ± SD, whereas the pooled results from three experiments are presented as Mean ± MAD

Table S12. Young's modulus (MPa) of the bilayer with 20% Cholesterol in the presence of Aβ42

Time (h)	Experiment 1	Experiment 2	Experiment 3	Mean ± MAD
**0**	37.97 ± 4.76	36.45 ± 5.64	41.35 ± 4.56	38.59 ± 1.84
**1**	38.55 ± 5.51	37.01 ± 6.43	41.98 ± 5.28	39.18 ± 1.86
**2**	38.04 ± 4.4	36.51 ± 4.56	41.43 ± 4.22	38.66 ± 1.84
**3**	32.61 ± 5.24	31.30 ± 7.54	35.51 ± 5.03	33.14 ± 1.58
**4**	26.92 ± 5.76	25.84 ± 4.34	29.31 ± 5.52	27.36 ± 1.30
**5**	21.49 ± 5.44	20.63 ± 6.76	23.40 ± 5.22	21.84 ± 1.04
**6**	17.42 ± 5.53	16.73 ± 5.23	18.97 ± 5.30	17.70 ± 0.84

Data from individual experiments are reported as Mean ± SD, whereas the pooled results from three experiments are presented as Mean ± MAD.

Table S13. Young's modulus (MPa) of the bilayer containing 20% cholesterol in the absence of Aβ42.

Time (h)	Experiment 1	Experiment 2	Experiment 3	Mean ± MAD
**0**	37.97 ± 5.23	41.91 ± 6.61	38.97 ± 6.21	40.26 ± 1.09
**1**	39.03 ± 5.68	40.98 ± 6.48	38.11 ± 6.09	39.37 ± 1.07
**2**	36.64 ± 5.94	38.47 ± 7.8	35.77 ± 7.33	36.96 ± 1.01
**3**	39.65 ± 3.92	41.63 ± 5.56	38.71 ± 5.22	40.01 ± 1.08
**4**	37.35 ± 5.96	39.21 ± 3.33	36.47 ± 3.13	37.67 ± 1.02
**5**	39.65 ± 5.98	41.63 ± 5.56	38.71 ± 5.22	40.01 ± 1.08
**6**	39.68 ± 4.56	41.63 ± 5.93	38.74 ± 5.57	40.03 ± 1.07

Data from individual experiments are reported as Mean ± SD, whereas the pooled results from three experiments are presented as Mean ± MAD

Table S14. Thickness (nm) of the POPC/POPS only bilayer in the presence of Aβ42

Time (h)	Experiment 1	Experiment 2	Mean ± MAD
**0**	3.57 ± 0.47	3.21 ± 0.51	3.39 ± 0.18
**3**	3.31 ± 0.86	3.48 ± 0.48	3.38 ± 0.08
**6**	3.63 ± 0.71	3.37 ± 0.36	3.50 ± 0.13

Data from individual experiments are reported as Mean ± SD, whereas the pooled results from three experiments are presented as Mean ± MAD

Table S15. Thickness (nm) of the POPC/POPS only bilayer in the presence of buffer (Control)

Time (h)	Experiment 1	Experiment 2	Mean ± MAD
**0**	3.71 ± 0.57	3.92 ± 0.48	3.81 ± 0.12
**3**	3.74 ± 0.44	4.08 ± 0.20	3.92 ± 0.17
**6**	3.70 ± 0.57	3.86 ± 0.29	3.78 ± 0.08

Data from individual experiments are reported as Mean ± SD, whereas the pooled results from three experiments are presented as Mean ± MAD

Table S16. Young's modulus (MPa) of the bilayer with 20% Cholesterol in the presence of MβCD

Time (h)	Experiment 1	Experiment 2	Experiment 3	Mean ± EM
**0**	35.39 ± 6.46	38.53 ± 4.05	37.61 ± 2.65	37.18 ± 1.19
**0.25**	18.88 ± 4.18	20.56 ± 6.26	20.06 ± 3.25	19.83 ± 0.63
**0.5**	18.58 ± 4.10	20.23 ± 3.97	19.74 ± 2.75	19.52 ± 0.62
**0.75**	17.20 ± 4.49	18.73 ± 4.35	18.28 ±2.38	18.08 ± 0.58
**1**	17.68 ± 3.69	19.26 ± 3.58	18.79 ± 4.25	18.58 ± 1.86
**2**	17.62 ± 4.32	19.19 ± 4.18	18.73 ± 3.64	18.51 ± 1.84
**3**	13.46 ± 2.42	14.65 ± 2.34	14.30 ± 4.42	14.14 ± 1.58
**4**	14.47 ± 2.79	15.75 ± 2.71	15.37 ± 4.02	15.20 ± 1.30
**5**	12.22 ± 3.29	13.30 ± 3.19	12.98 ± 4.12	12.83 ± 1.04
**6**	10.21 ± 2.69	11.12 ± 2.61	10.58 ± 5.36	10.52 ± 0.84

Data from individual experiments are reported as Mean ± SD, whereas the pooled results from three experiments are presented as Mean ± MAD

Table S17. Thickness (nm) of the bilayer with 20% Cholesterol in the presence of MβCD

Time (h)	Experiment 1	Experiment 2	Mean ± MAD
**0**	4.35 ± 0.74	4.66 ± 0.62	4.50 ± 0.16
**3**	3.29 ± 0.26	3.82 ± 0.45	3.55 ± 0.26
**6**	2.88 ± 0.52	3.12 ± 0.56	3.00 ± 0.12

Data from individual experiments are reported as Mean ± SD, whereas the pooled results from three experiments are presented as Mean ± MAD

Table S18: Maxima values of fluorescence intensity (a.u.) recorded at each time point for PC:PS 20% NBD Chol bilayer incubated with 50 nM Aβ42

Time (h)	Experiment 1	Experiment 2	Experiment 3	Mean ± MAD
0	45.60	38.88	42.25	42.24 ± 2.24
1	94.13	132.14	103.13	109.8 ± 14.89
2	184.24	145.75	164.99	164.99 ± 12.83
3	201.62	201.60	208.58	203.93 ± 3.09
4	227.64	245.78	236.71	236.71 ± 6.04
5	375.03	401.95	388.49	388.49 ± 8.97
6	500.96	519.94	510.45	510.45 ± 6.32

Table S19: Maxima values of fluorescence intensity (a.u.) recorded at each time point for PC:PS 20% NBD Chol bilayer incubated with 3 mM MβCD

Time (h)	Experiment 1	Experiment 2	Experiment 3	Mean ± MAD
0	48.36	49.88	49.12	49.12 ± 0.50
1	529.66	524.85	527.26	527.25 ± 1.60
2	529.85	535.32	532.59	532.58 ± 1.82
3	771.61	782.23	776.92	776.92 ± 3.54
4	720.53	755.55	738.04	738.04 ± 11.67
5	758.84	777.40	768.12	768.12 ± 6.18
6	782.57	770.36	776.47	776.46 ± 4.07

Table S20: Maxima values of fluorescence intensity (a.u) recorded at each time point for PC:PS 20% NBD Chol bilayer incubated with 10 mM HEPES (control experiment)

Time (h)	Experiment 1	Experiment 2	Experiment 3	Mean ± MAD
0	46.15	39.56	42.85	42.85 ± 2.19
1	44.46	39.13	41.80	41.79 ± 1.77
2	49.48	61.58	55.53	55.53 ± 4.03
3	49.96	72.94	61.45	61.45 ± 7.66
4	103.13	77.93	90.54	90.53 ± 8.40
5	104.51	94.48	99.49	99.49 ± 3.34
6	119.53	134.13	126.83	126.83 ± 4.86

Table S21. Young's modulus (MPa) of the bilayer with 20% 25-NBD Cholesterol in the presence of Aβ42

Time (h)	Experiment 1	Experiment 2	Experiment 3	Mean ± MAD
**0**	42.33 ± 4.10	37.53 ± 4.86	45.03 ± 4.59	41.63 ± 2.73
**1**	41.13 ± 5.01	36.47 ± 5.3	43.76 ± 5.01	40.45 ± 2.65
**2**	32.13 ± 3.37	32.63 ± 3.57	39.15 ± 3.37	36.19 ± 2.37
**3**	30.07 ± 3.36	30.07 ± 3.17	36.08 ± 3.15	33.35 ± 2.19
**4**	27.92 ± 2.99	27.92 ± 2.82	33.50 ± 2.82	30.97 ± 2.03
**5**	23.04 ± 3.44	23.04 ± 3.25	27.64 ± 3.25	25.55 ± 1.67
**6**	19.32 ± 2.81	19.32 ± 2.65	23.18 ± 2.65	21.43 ± 1.40

Data from individual experiments are reported as Mean ± SD, whereas the pooled results from three experiments are presented as Mean ± MAD.

Table S22. Thickness (nm) of the bilayer with 25-NBD cholesterol in the presence of 50 nM Aβ42

Time (h)	Experiment 1	Experiment 2	Mean ± MAD
**0**	5.06 ± 0.25	4.78 ± 0.71	4.92 ± 0.14
**3**	4.08 ± 0.22	3.93 ± 0.48	4.05 ± 0.07
**6**	2.90 ± 0.24	3.12 ± 0.53	3.01 ± 0.11

Data from individual experiments are reported as Mean ± SD, whereas the pooled results from three experiments are presented as Mean ± MAD

Statistical Analysis Report

Figure 2B

PC:PS

t-Test: Two-Sample Assuming Unequal Variances

	0 hour	6 hour
Mean	20.23666667	17.57333333
Variance	0.166233333	0.024433333
Observations	3	3
Hypothesized Mean Difference	0	
df	3	
t Stat	10.56449556	
P(T<=t) one-tail	0.090585881	
t Critical one-tail	2.353363435	
P(T<=t) two-tail	0.196171762	
t Critical two-tail	3.182446305	

PC:PS:10% Chol

t-Test: Two-Sample Assuming Unequal Variances

	0 hour	6 hour
Mean	29.29333333	17.71666667
Variance	6.557033333	4.212433333
Observations	3	3
Hypothesized Mean Difference	0	
df	4	
t Stat	6.110082268	
P(T<=t) one-tail	0.018159459	
t Critical one-tail	2.131846786	
P(T<=t) two-tail	0.027318919	
t Critical two-tail	2.776445105	

PC:PS:20% Chol

t-Test: Two-Sample Assuming Unequal Variances

	0 hour	6 hour
Mean	39.41666667	17.89333333
Variance	1.289633333	0.212433333
Observations	3	3
Hypothesized Mean Difference	0	
df	3	
t Stat	30.41764274	
P(T<=t) one-tail	0.003902805	
t Critical one-tail	2.353363435	
P(T<=t) two-tail	0.00610561	
t Critical two-tail	3.182446305	

PC:PS:30% Chol

t-Test: Two-Sample Assuming Unequal Variances

	0 hour	6 hour
Mean	51.77333333	21.08666667
Variance	1.382433333	5.706133333
Observations	3	3
Hypothesized Mean Difference	0	
df	3	
t Stat	19.96324465	
P(T<=t) one-tail	0.00137353	
t Critical one-tail	2.353363435	
P(T<=t) two-tail	0.00274706	
t Critical two-tail	3.182446305	

Figure 3C

t-Test: Two-Sample Assuming Unequal Variances

	0 hour	3 hour
Mean	4.325	3.66
Variance	0.00125	0.0072
Observations	2	2
Hypothesized Mean Difference	0	
df	1	
t Stat	10.23076923	
P(T<=t) one-tail	0.051014476	
t Critical one-tail	6.313751515	
P(T<=t) two-tail	0.042028953	
t Critical two-tail	12.70620474	

t-Test: Two-Sample Assuming Unequal Variances

	0 hour	6 hour
Mean	4.325	3.265
Variance	0.00125	5E-05
Observations	2	2
Hypothesized Mean Difference	0	
df	1	
t Stat	41.57662065	
P(T<=t) one-tail	0.004654507	
t Critical one-tail	6.313751515	
P(T<=t) two-tail	0.007309014	
t Critical two-tail	12.70620474	

Figure 3D

t-Test: Two-Sample Assuming Unequal Variances

	0 hour	0 hour
Mean	39.61666667	38.59
Variance	4.194533333	6.2908
Observations	3	3
Hypothesized Mean Difference	0	
df	4	
t Stat	0.549160091	
P(T<=t) one-tail	0.30605585	
t Critical one-tail	2.131846786	
P(T<=t) two-tail	0.6121117	
t Critical two-tail	2.776445105	

t-Test: Two-Sample Assuming Unequal Variances

	1 hour	1 hour
Mean	39.37333333	39.18
Variance	2.147633333	6.4729
Observations	3	3
Hypothesized Mean Difference	0	
df	3	
t Stat	0.114051315	
P(T<=t) one-tail	0.458200845	
t Critical one-tail	2.353363435	
P(T<=t) two-tail	0.916401691	
t Critical two-tail	3.182446305	

t-Test: Two-Sample Assuming Unequal Variances

	2 hour	2 hour
Mean	36.96	38.84333333
Variance	1.8993	7.964233333
Observations	3	3
Hypothesized Mean Difference	0	
df	3	
t Stat	-1.038655588	
P(T<=t) one-tail	0.187662575	
t Critical one-tail	2.353363435	
P(T<=t) two-tail	0.37532515	
t Critical two-tail	3.182446305	

t-Test: Two-Sample Assuming Unequal Variances

	3 hour	3 hour
Mean	39.99666667	33.14
Variance	2.221733333	4.6417
Observations	3	3
Hypothesized Mean Difference	0	
df	4	
t Stat	4.533179901	
P(T<=t) one-tail	0.005275875	
t Critical one-tail	2.131846786	
P(T<=t) two-tail	0.03055175	
t Critical two-tail	2.776445105	

t-Test: Two-Sample Assuming Unequal Variances

	4 hour	4 hour
Mean	37.67666667	27.35666667
Variance	1.956933333	3.153233333
Observations	3	3
Hypothesized Mean Difference	0	
df	4	
t Stat	7.907201248	
P(T<=t) one-tail	0.006919677	
t Critical one-tail	2.131846786	
P(T<=t) two-tail	0.023839354	
t Critical two-tail	2.776445105	

t-Test: Two-Sample Assuming Unequal Variances

	5 hour	5 hour
Mean	39.99666667	21.84
Variance	2.221733333	2.0101
Observations	3	3
Hypothesized Mean Difference	0	
df	4	
t Stat	15.28735921	
P(T<=t) one-tail	0.000533953	
t Critical one-tail	2.131846786	
P(T<=t) two-tail	0.003167906	
t Critical two-tail	2.776445105	

t-Test: Two-Sample Assuming Unequal Variances

	6 hour	6 hour
Mean	40.01666667	17.70666667
Variance	2.173033333	1.316033333
Observations	3	3
Hypothesized Mean Difference	0	
df	4	
t Stat	20.68738342	
P(T<=t) one-tail	0.000161274	
t Critical one-tail	2.131846786	
P(T<=t) two-tail	0.002432255	
t Critical two-tail	2.776445105	

Figure 4D

t-Test: Two-Sample Assuming Unequal Variances

	0 hour	*3 hour*
Mean	4.505	3.555
Variance	0.04805	0.14045
Observations	2	2
Hypothesized Mean Difference	0	
df	2	
t Stat	3.094446124	
P(T<=t) one-tail	0.004524107	
t Critical one-tail	2.91998558	
P(T<=t) two-tail	0.042482143	
t Critical two-tail	4.30265273	

t-Test: Two-Sample Assuming Unequal Variances

	0 hour	*6 hour*
Mean	4.505	3
Variance	0.04805	0.0288
Observations	2	2
Hypothesized Mean Difference	0	
df	2	
t Stat	7.677672129	
P(T<=t) one-tail	0.000827233	
t Critical one-tail	2.91998558	
P(T<=t) two-tail	0.008654466	
t Critical two-tail	4.30265273	

Figure 5D

t-Test: Two-Sample Assuming Unequal Variances

	Control	*Ab42*
Mean	126.83	510.45
Variance	53.29	90.0601
Observations	3	3
Hypothesized Mean Difference	0	
df	4	
t Stat	-55.49615165	
P(T<=t) one-tail	1.56E-04	
t Critical one-tail	2.131846786	
P(T<=t) two-tail	0.004285951	
t Critical two-tail	2.776445105	

t-Test: Two-Sample Assuming Unequal Variances

	Control	*MβCD*
Mean	126.83	776.4666667
Variance	53.29	37.27103333
Observations	3	3
Hypothesized Mean Difference	0	
df	4	
t Stat	-118.2389248	
P(T<=t) one-tail	1.53E-05	
t Critical one-tail	2.131846786	
P(T<=t) two-tail	9.47E-04	
t Critical two-tail	2.776445105	

Figure 6C

t-Test: Two-Sample Assuming Unequal Variances

	1 hour	*6 hour*
Mean	40.45333333	20.60666667
Variance	13.62943333	4.966533333
Observations	3	3
Hypothesized Mean Difference	0	
df	3	
t Stat	7.971477478	
P(T<=t) one-tail	0.002059451	
t Critical one-tail	2.353363435	
P(T<=t) two-tail	0.004118902	
t Critical two-tail	3.182446305	

Figure 6D

t-Test: Two-Sample Assuming Unequal Variances

	0 hour	*3 hour*
Mean	4.92	4.02
Variance	0.0196	0.0063
Observations	3	3
Hypothesized Mean Difference	0	
df	3	
t Stat	9.686196045	
P(T<=t) one-tail	0.011683234	
t Critical one-tail	2.353363435	
P(T<=t) two-tail	0.048366467	
t Critical two-tail	3.182446305	

t-Test: Two-Sample Assuming Unequal Variances

	0 hour	*6 hour*
Mean	4.92	3.010333333
Variance	0.0196	0.012210333
Observations	3	3
Hypothesized Mean Difference	0	
df	4	
t Stat	18.54530947	
P(T<=t) one-tail	0.004487787	
t Critical one-tail	2.131846786	
P(T<=t) two-tail	0.009287787	
t Critical two-tail	2.776445105	
